# Occupational risk factors for depression and anxiety symptoms: Insights from a large cohort study during and after the SARS-CoV-2 pandemic

**DOI:** 10.1371/journal.pone.0346871

**Published:** 2026-04-15

**Authors:** Swaantje Casjens, Jan Hovanec, Nadine Glaser, Janka Massag, Laura Pfrommer, Nils Opel, André Karch, Saskia Muellmann, Irene Moor, Michael Gekle, Matthias Girndt, Simone Hettmer, Jessica I. Höll, Michael Heuser, Thomas Frese, Rafael Mikolajczyk, Thomas Behrens

**Affiliations:** 1 Ruhr University Bochum, Medical Faculty, Institute for Prevention and Occupational Medicine of the German Social Accident Insurance, Institute of the Ruhr University Bochum (IPA), Bochum, Germany; 2 Institute for Medical Epidemiology, Biometrics, and Informatics (IMEBI), Interdisciplinary Centre for Health Sciences, Medical Faculty of the Martin Luther University Halle-Wittenberg, Halle (Saale), Germany; 3 German Center for Mental Health, Site Halle-Jena-Magdeburg, Halle (Saale), Germany; 4 Department of Psychiatry & Neuroscience, Campus Benjamin Franklin, Charité Universitätsmedizin Berlin, Berlin, Germany; 5 Institute of Epidemiology and Social Medicine, University of Münster, Münster, Germany; 6 Leibniz Institute for Prevention Research and Epidemiology - BIPS, Bremen, Germany; 7 Leibniz ScienceCampus Digital Public Health Bremen (LSC DiPH), Bremen, Germany; 8 Martin Luther University Halle-Wittenberg, Medical Faculty, Interdisciplinary Centre for Health Sciences (PZG), Institute of Medical Sociology, Halle (Saale), Germany; 9 Julius-Bernstein-Institute of Physiology, Medical Faculty of the Martin Luther University Halle-Wittenberg, Halle (Saale), Germany; 10 Department of Internal Medicine II, Martin Luther University Halle-Wittenberg, Halle (Saale), Germany; 11 Paediatric Haematology and Oncology, Martin Luther University Halle-Wittenberg, Halle (Saale), Germany; 12 Department of Internal Medicine IV, University Hospital Halle (Saale), Martin Luther University Halle-Wittenberg, Halle (Saale), Germany; 13 Institute of General Practice and Family Medicine, Interdisciplinary Centre of Health Sciences, Medical Faculty of the Martin Luther University Halle-Wittenberg, Halle (Saale), Germany; Johns Hopkins Bloomberg School of Public Health: Johns Hopkins University Bloomberg School of Public Health, UNITED STATES OF AMERICA

## Abstract

**Objectives:**

An increased risk for an occupation-related SARS-CoV-2 infection has been linked to higher psychological distress. This online survey investigates the prevalence of depressive and anxiety symptoms in a large sample of 34,303 participants from the German cohort for digital health research (DigiHero) after the pandemic (late 2023, t1) and retrospectively from the Omicron wave (early 2022, t0), emphasizing variations across occupational groups and work-related risk factors.

**Methods:**

Participants reported their employment status (currently working; seeking employment; not working). Workers provided their primary occupation to assess occupational SARS-CoV-2 infection risk. Symptoms of depression and anxiety (assessed via PHQ-4) and additional occupational risk factors were solicited for each time point. Associations between occupational exposure and stressors with the four-level PHQ-4 outcome were analyzed separately for t1 and t0 using ordinal regression and expressed as odds ratios (OR) with 95% confidence intervals (CI).

**Results:**

Over 60% of respondents were working at t1, and 1.4% classified themselves as seeking a job. Job seekers reported highest and non-working individuals lowest depressive and anxiety symptoms. Symptom severity varied by occupation with elevated odds in traffic/logistics professions exclusively at t1 (OR=1.24, 95% CI 1.04–1.48) and healthcare professions exclusively at t0 (OR=1.08, 95% CI 1.01–1.16). High occupational SARS-CoV-2 infection risk was linked to symptoms at t0. Overall, these associations were modest and partly attenuated after additional adjustment for individual work-related stressors (e.g., loneliness at work, chronic work-related stress, work-privacy conflicts). At both timepoints, individual stressors and sociodemographic factors showed stronger associations with severe symptoms than occupation (e.g., chronic work-related stress at t1 OR=2.87, 95% CI 2.70–3.04).

**Conclusions:**

Persistent post-pandemic depressive and anxiety symptoms among workers emphasize the importance of addressing individual psychosocial work-related stressors.

## Introduction

By the end of the pandemic, over 765 million infections and 6.9 million deaths were registered worldwide [1]. Generally, workplaces are considered high-risk environments for transmission, primarily due to proximity and frequent contacts with colleagues, clients, or patients. Early in the pandemic, it became clear that the risk of SARS-CoV-2 infection varied across occupational groups. Workers in essential occupations were particularly vulnerable, with healthcare professionals facing the highest risk of infection [2–4], but risks were also elevated for transportation, food industry, safety and security, as well as workers under precarious working conditions [5]. In later waves of the pandemic, when schools and businesses reopened, workers in occupations like teaching, social care, or food preparation were also at greater risk [6,7]. SARS-CoV-2 infections could not only lead to physical strain but also have a direct impacted on mental health, presenting with acute symptoms such as delirium and post-illness sequelae including depression, anxiety, and post-traumatic stress disorder [8]. Furthermore, the mental health burden was exacerbated by public health measures [9], fear of infection [10], loneliness [11], and job insecurity or financial concerns [12].

Since the onset of the pandemic, numerous studies have examined psychological strain across various occupational sectors, with particular emphasis on healthcare workers [13,14]. Occupational infection risk negatively impacts mental health by exposing workers in high-risk environments to heightened stress and fear of infection, while factors such as specific job roles, limited specialized training or isolation further exacerbate mental health challenges [15]. However, some studies found no evidence of increased depressive or anxiety symptoms among healthcare workers compared to the general population or workers in non-essential occupations [16,17]. During the pandemic, social workers showed high levels of distress and depression, while increased symptoms were also observed among other occupational groups, including workers in utility, food supply, and transportation [16], grocery store workers [18], social workers [19], or teachers [20] in comparison to either non-essential workers or the general population.

Other studies investigated the impact of working in industries associated with higher SARS-CoV-2 risk on mental health. An analysis of SHARE (Survey of Health, Ageing and Retirement in Europe) data from 2020, covering workers aged 50–70, found that workers in high-risk sectors (i.e., health, social work, education, hotels, and restaurants) experienced pronounced declines in mental health. This was particularly seen in women, suggesting the presence of sex-based disparities [21]. A study of non-healthcare workers in Germany conducted between December 2020 and June 2021 similarly found an association between increased symptoms of depression and anxiety and occupational SARS-CoV-2 infection risk [22]. A follow-up in late 2022 indicated that the impact of occupational SARS-CoV-2 infection risk, along with other occupational factors such as work-privacy conflicts, chronic work-related stress, and overcommitment on psychological distress remained high [23].

While some evidence is available regarding occupational risk and depressive symptoms throughout the pandemic, studies specifically addressing the late pandemic and post-pandemic periods remain limited. Hence, this article investigates the prevalence of depressive and anxiety symptoms among German citizens in the post-pandemic period (late 2023) and during the SARS-CoV-2 Omicron wave (early 2022, retrospectively). This analysis examines changes in mental health across occupations and sectors over time, focusing on occupations classified as high-risk for viral transmission and additional occupational risk factors for depressive and anxiety symptoms, such as personal customer contact, chronic work-related stress, overcommitment to work, or work-privacy conflicts.

## Materials and methods

### DigiHero study and data assessment

We analyzed data from the ongoing cohort study on digital health research in Germany (DigiHero, DRKS Registration-ID: DRKS00025600). DigiHero was initiated in January 2021 in Halle (Saale) and has since then expanded to a nationwide digital cohort study with currently more than 120,000 participants. Addresses for approximately one-third of households were randomly selected from resident registries across Germany. Residents aged 18 and older were invited by letter. In cities with populations over 100,000 inhabitants, the invitation sample was limited to 30,000 individuals. Regional response proportions ranged from two to eight percent. Additionally, all German residents aged 18 and older could self-register via the DigiHero website, resulting in a convenience sample based on voluntary participation. Participation is fully digital, using the LimeSurvey® platform, with four scheduled contacts annually.

On November 3, 2023, 81,498 DigiHero participants were invited via e-mail to take part in a survey examining employment, work conditions, and well-being (‘Work and Well-Being’ questionnaire, see supplement). Through December 15, 2023, 42,577 participants completed the questionnaire (52% response).

All participants provided written electronic informed consent by actively selecting designated checkboxes confirming informed consent and agreement to the privacy policy within the online survey. Ethical approval from the Ethics Committee of the Martin Luther University Halle-Wittenberg, Germany, was obtained for the DigiHero study and the ‘Work and Well-Being’ questionnaire (Reg. No. 2020−076). We followed STROBE reporting guidelines for observational studies (S1 File).

The ‘Work and Well-Being’ questionnaire captured professional activity (currently working; seeking employment; not working), psychological and occupational stress at the current time (t1), and a retrospective assessment of the Omicron wave (t0). Currently working participants answered additional questions regarding their primary occupation; multiple occupations were noted. Participants selected their current occupation from a drop-down menu based on the hierarchical structure of the five-digit coding scheme German Classification of Occupations (Klassifikation der Berufe (KldB), [24]). This survey was limited to the third hierarchical level, comprising 10 occupational areas (1-digit KldB), 37 occupational main groups (2-digit KldB), and 144 occupational groups (3-digit KldB). Furthermore, the 37 occupational main groups were aggregated in five occupational sectors and 14 occupational segments. Additionally, participants’ current job titles were obtained. Based on power calculations analogous to a study from the UK Biobank [25], 353 randomly selected job titles were subjected to independent re-coding by an expert to assess the reliability of participants’ self-coded occupations. Cohen’s Kappa statistic indicated comparable reliability to the UK study (1-digit KldB κ = 0.63, 2-digit KldB κ = 0.55, 3-digit KldB κ = 0.48).

Occupational SARS-CoV-2 infection risk was assigned based on the subjects’ occupational information. In general, workers having contact with SARS-CoV-2-infected individuals at work face a significantly elevated risk [26]. Therefore, workers in medical and non-medical healthcare occupations, including healthcare support, cleaning in healthcare facilities or retail sales of medical and health products, as well as those in theology and church community services, were classified as having a very high occupational risk. The assignment of non-healthcare workers into high or probable risk categories followed a previous study’s methodology [22], which was primarily based on whether the occupation involved frequent and likely contact with the public during professional activities (high risk) or probable contact (probable risk). The majority of participants in the high-risk group were employed in education, social work, law enforcement, correctional services, grocery retail, gastronomy, postal and delivery services, transportation, and cleaning services. Those at probable occupational infection risk included employees in public administration, financial services, sales, and occupational safety and health. All other occupations were not assigned an elevated risk of occupational SARS-CoV-2 infection (classified as “none”).

Additionally, status of employment (permanent employee with no predefined end date of employment; fixed-term employee; temporary/agency worker; civil servant; self-employed/freelancer) and job duration in years were recorded. If participants reported different occupations at t0 and t1, their t0 job titles were also recorded. Weekly working hours (full-time ≥35 hours; 30–35 hours; 20- < 30 hours; < 20 hours), and frequency of personal customer contact (daily; occasionally; rarely; none) were recorded for both time points.

Psychological and occupational stress were assessed with validated instruments. The brief four-item Patient Health Questionnaire (PHQ-4) [27], comprising the two-item PHQ-2 and the two-item Generalized Anxiety Disorder (GAD)-2 scales, was used to rate depressive and anxiety symptoms. PHQ-4 scores were categorized as normal (0–2), mild (3–5), moderate (6–8), and severe (9–12) symptoms. Work-privacy conflicts were rated analogously to the German version of the Copenhagen Psychosocial Questionnaire using the question: “To what extent do the demands of your work interfere with your private and family life?” [28]. The middle category “somewhat” served as reference against high (”to a very large or large extent”) and low (“to a small or very small extent”) work-privacy conflicts. Loneliness at work was assessed using the three-item UCLA Loneliness Scale [29] with a workplace-specific focus. For example, the first adapted item was: ”I lack companionship at work.” The three questions on loneliness utilized a 3-point Likert scale (often = 3, sometimes = 2, rarely or never = 1), allowing the total score to range between 3 and 9. A score of 6 or higher indicated moderate to severe loneliness at work [30].

Chronic work-related stress was assessed using the short version of the effort-reward imbalance (ERI) questionnaire comprising three effort and seven reward items [31]. A higher effort sum score indicates that more demands are perceived as stressful, while a higher reward sum score reflects a greater level of reward. Therefore, an effort-reward ratio close to zero suggests a favorable state (i.e., relatively low effort and relatively high reward), whereas values greater than 1 indicate a high level of effort that is not adequately compensated by the rewards received or expected. Intrinsic effort was assessed as overcommitment to work using the six-item overcommitment questionnaire [32]. The overcommitment score ranges from 6 to 24, with higher scores indicating more likely excessive engagement. As both instruments capture long-term parameters, these two instruments were inquired exclusively at t1, unlike the other measures, which were assessed at both t0 and t1.

Sociodemographic characteristics of the study population, including age, sex (male; female; other), and education (low: ≤ 10 years of schooling; medium: > 10 years of schooling; high: university degree) were obtained during the initial recruitment between January 21, 2021 and December 12, 2023. S2 File provides a summary of the study variables from the ‘Work and Well-Being’ questionnaire and the demographic characteristics used in the analyses.

### Study sample

For the current analysis, eligibility was restricted to participants with complete PHQ-4 scores for t0 and t1, employment status data, and not enrolled as students. From the currently working individuals, participants were excluded due to missing or insufficient job titles (n = 478), employees on temporary leave (n = 9), career changers (n = 1,847), and those without information on their employment status at t0 (n = 978). As a result, data from 34,303 subjects were analyzed.

### Statistical analysis

Continuous variables were characterized by median and interquartile range (IQR) or mean and standard deviation. Categorical variables were summarized by frequencies and percentages. The outcome, psychological distress, as assessed using the four-category PHQ-4 variable, was analyzed with ordinal regression (proportional odds models), and results reported as odds ratios (OR) with 95% confidence intervals (95% CI) for t0 and t1. Models with occupational sector, segment, and main group as risk factors present ORs for each category, with reference to all other occupations. For other factors, reference categories are specified. A directed acyclic graph (DAG, http://www.dagitty.net/) was used to determine minimally sufficient adjustment sets of covariates to estimate total effects (TE) of occupational exposure on depressive and anxiety symptoms (S3 File). Accordingly, controlling for sex, age, education, employment status, and weekly working hours adequately accounts for confounding to estimate TE. Further adjustments for work-related psychosocial stressors, including chronic work-related stress, loneliness at work, overcommitment to work, and work-privacy conflicts, were applied to reveal controlled direct effect (CDE) estimates. Analyses were stratified by sex, age group, and educational attainment. Sensitivity analyses were conducted by combining the very high and high occupational SARS-CoV-2 risk categories to address potential misclassification arising from changing risks at different pandemic time points. All models were analyzed using complete-case analysis. Statistical analyses were performed in R (Version 4.5.0) using the ‘ordinal’ package. Sankey graphs were prepared with SankeyMATIC.

## Results

### Study population

Table 1 depicts the characteristics of the overall study population. Over 60% were employed at the time of the survey, whereas only 1.4% identified themselves as job seekers. Most non-working participants were of retirement age (median 68 years, IQR 64–73 years). A notably higher proportion of women than men participated in the survey (59.6% vs. 39.2%), along with 39 individuals identifying as ’other’. More than 60% of the participants held a university degree.

**Table 1:** Sample description of all study participants according to depression and anxiety symptoms and results of univariable proportional odds models at t0 and t1 (N = 34.303).

	N	%	PHQ-4 (t1, November 2023)				PHQ-4 (t0, Omicron wave 2022)			
			Med. (IQR)	Mean (Std)	OR^a^	95% CI	Med. (IQR)	Mean (Std)	OR^a^	95% CI
Total	34,303		2 (1–4)	2.73 (2.50)			2 (1–4)	2.63 (2.55)		
Professional activity										
Job seeker	486	1.4	4 (2–6)	4.06 (2.86)	2.23	1.89 - 2.64	3 (1–5)	3.62 (3.01)	1.62	1.37 - 1.92
Not working	12,869	37.5	2 (1–4)	2.45 (2.42)	0.74	0.70 - 0.77	1 (0-3)	2.09 (2.34)	0.51	0.49 - 0.53
Working (ref.)	20,948	61.1	2	2.87 (2.51)	1		3 (1–4)	2.94 (2.61)	1	
Educational attainment										
Low: ≤ 10y schooling	449	1.3	3 (1–5)	3.70 (3.05)	2.15	1.80 - 2.57	3 (1–5)	3.51 (3.10)	1.90	1.59 - 2.27
Medium: > 10y schooling	11,656	34.0	3 (1–4)	3.01 (2.64)	1.39	1.33 - 1.45	2 (1–4)	2.90 (2.71)	1.35	1.30 - 1.41
High: university degree (ref.)	21,866	63.7	2 (1–4)	2.56 (2.38)	1		2 (0-4)	2.46 (2.43)	1	
Missing	332	1.0	3 (1–4)	3.27 (2.82)	–		3 (1–4)	3.16 (2.85)	–	
Sex										
Female	20,446	59.6	3 (1–4)	3.04 (2.55)	1.84	1.76 - 1.92	3 (1–4)	3.04 (2.63)	2.21	2.11 - 2.31
Other	39	0.1	5 (3–8)	5.69 (3.46)	8.98	4.97 - 16.22	5 (3–7)	5.49 (3.39)	9.84	5.48 - 17.66
Male (ref.)	13,457	39.2	2 (0-3)	2.24 (2.32)	1		1 (0-3)	1.99 (2.28)	1	
Missing	361	1.1	3 (1–4)	3.20 (2.82)	–		3 (1–4)	3.06 (2.90)	–	
Age [years] (Median, range)	33,883	57 (19-91)			0.81^b^	0.79 - 0.82			0.73^b^	0.72 - 0.75

a Unadjusted estimates from proportional odds models assessing the risk of elevated depressive and anxiety symptoms (based on PHQ-4 categories).

b Odds ratio and 95% CI per 10 years of age.

*PHQ-4*, brief 4-item Patient Health Questionnaire to assess depression and anxiety symptoms; *t1*, November 2023; *t0*, Omicron wave of 2022, retrospectively; *Med*, median; *IQR*, interquartile range; *Std*, standard deviation; *OR*, odds ratio; *95% CI*, 95% confidence interval; *ref*, reference.

**Fig 1:**
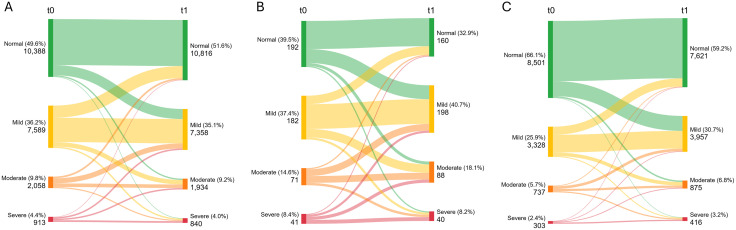
Mental distress in early 2022 (t0, retrospectively assessed) and late 2023 (t1) measured with the 4-item Patient Health Questionnaire (PHQ-4) among workers (A), job seekers (B), and non-working participants (C).

After the pandemic (t1), the median PHQ-4 score was 2 (IQR 1−4), with 4% of participants experiencing severe (PHQ-4 ≥ 9) and 13% substantial (PHQ-4 ≥ 6) symptoms of depression and anxiety. Symptom severity reported retrospectively for the Omicron wave (t0) were similar (Table 1). The proportion of participants with more severe symptoms was lowest among non-working subjects, followed by currently working participants. Job seekers reported the most severe symptoms (Fig 1). This is further demonstrated by the comparison with working participants, showing elevated odds at t1 (OR=2.23, 95% CI 1.89–2.64) and at t0 (OR=1.62, 95% CI 1.37–1.92). While symptoms improved among working and non-working participants post-pandemic, the proportion of distressed job seekers increased from 60.9% to 67.1%. Other risk factors for more severe symptoms included lower educational attainment, female sex, and identifying as ‘other’ sex (the latter based on a very small sample). In contrast, increasing age conferred a protective effect (Table 1 and S4 File).

Among working participants (n = 20,948), the proportion of individuals with high work-privacy conflicts or feeling lonely at work decreased, whereas personal customer contact increased after the pandemic. Most working subjects were permanently employed (78.1%). Potentially more stressful employment states, such as fixed-term or temporary employment, or multiple jobs were reported by less than 5%. Overall, 3,479 workers (16.6%) were classified as having a very high occupational SARS-CoV-2 infection risk, 1,585 workers (7.6%) as having high risk, and 2,571 workers (12.3%) as having probable risk (Table 2).

**Table 2:** Description of occupational characteristics and strain among 20,948 workers.

		t1 (November 2023)		t0 (Omicron wave 2022)	
		N	%	N	%
Employment status	Permanent	16,369	78.1	16,369	78.1
	Fixed-term	847	4.0	847	4.0
	Temporary	36	0.2	36	0.2
	Civil servant	2,062	9.8	2,062	9.8
	Self-employed	1,499	7.2	1,499	7.2
	Missing	135	0.6	135	0.6
Job duration [years]	Median (IQR)	20,878	18 (8-29)		
Professional activity	One job	19,974	95.4		
	Multiple jobs	974	4.6		
Occupational SARS-CoV-2 infection risk	None	13,313	63.6	13,313	63.6
	Probable	2,571	12.3	2,571	12.3
	High	1,585	7.6	1,585	7.6
	Very high	3,479	16.6	3,479	16.6
Personal customer contact	Yes, daily	10,106	48.2	7,790	37.2
	Yes, occasionally	3,986	19.0	3,582	17.1
	Yes, rarely	3,082	14.7	3,779	18.0
	None	3,675	17.5	5,653	27.0
	Missing	99	0.5	144	0.7
Weekly working hours	Median (IQR)	20,467	38.5 (32–40)	20,560	39 (32–40)
	Full time, ≥ 35h	14,438	68.9	14,619	69.8
	30-35h	2,866	13.7	2,702	12.9
	20- < 30h	2,184	10.4	2,203	10.5
	<20h	979	4.7	1,034	4.9
	Missing	481	2.3	390	1.9
Loneliness at work	Moderate/severe	1,592	7.6	4,222	20.2
	None/mild	19,066	91.0	16,458	78.6
	Missing	290	1.4	268	1.3
Chronic work-related stress	Yes	12,835	61.3		
	No	7,682	36.7		
	Missing	431	2.1		
Over-commitment to work [6–24]	Median (IQR)	20,837	15 (12–18)		
Work-privacy conflicts	High	2,764	13.2	4,590	21.9
	Medium	5,887	28.1	5,871	28.0
	Low	12,255	58.5	10,452	49.9
	Missing	42	0.2	35	0.2

*IQR*, interquartile range.

### Risk assessment of depressive and anxiety symptoms by occupational classification

Risk factors for more severe depressive and anxiety symptoms observed in the overall study population were also present among working participants. These included younger age, being female, and lower educational attainment (Table S5.1 in S5 File). Furthermore, employees with fixed-term or temporary contracts were at higher risk compared to permanent employees at both time points, as were part-time workers.

Occupations with a higher risk of more severe depressive and anxiety symptoms after the pandemic, compared to before, include those in traffic and logistics without vehicle driving (t1: OR=1.24, 95% CI 1.04–1.48; t0: OR=1.04, 95% CI 0.87–1.24, Table 3). At t0, an increased risk was observed in the personal services sector (OR=1.08, 95% CI 1.02–1.15), which was not elevated at t1 (OR=0.95, 95% CI 0.89–1.00). The effect was especially seen in medical and health care professionals (t0: OR=1.08, 95% CI 1.01–1.16; t1: OR=0.88, 95% CI 0.82–0.95) but attenuated after additional adjustment for occupational factors (CDE, Table S5.2 in S5 File). Occupations with consistently elevated odds, e.g., software development and programming, public administration, management assistants in transport and logistics) or reduced odds (e.g., professionals in human medicine and dentistry, teaching and training professionals), irrespective of time, were primarily observed in the CDE models or at occupational group level (Tables S5.2 and S5.3 in S5 File).

**Table 3:** Modeling the risk of elevated depressive and anxiety symptoms (PHQ-4 categories) among 20,948 working participants by occupational sector, segment and main group.

KldB	Occupations	t1 (November 2023)			t0 (Omicron wave, 2022)		
		N	OR^a^	95% CI	N	OR^a^	95% CI
**S1**	**Production of goods**	2,892	1.03	0.95-1.11	2,909	0.93	0.86-1.01
**S11**	**Agriculture, forestry and horticulture**	297	1.13	0.91-1.41	302	1.12	0.91-1.39
11	Occupations in agriculture, forestry, and farming	214	1.05	0.81-1.36	218	1.11	0.86-1.43
12	Occupations in gardening and floristry	83	1.36	0.90-2.05	84	1.17	0.78-1.73
**S12**	**Manufacturing**	610	1.16	0.99-1.35	616	1.08	0.92-1.26
21	Occupations in production and processing of raw materials, glass- and ceramic-making and -processing	56	0.95	0.57-1.59	56	1.08	0.65-1.80
22	Occupations in plastic-making and -processing, and wood-working and -processing	130	0.95	0.68-1.32	130	0.87	0.61-1.22
23	Occupations in paper-making and -processing, printing, and in technical media design	52	1.37	0.81-2.32	52	1.61	0.96-2.71
24	Occupations in metal-making and -working, and in metal construction	285	1.21	0.96-1.51	289	1.06	0.84-1.33
28	Occupations in textile- and leather-making and -processing	30	1.40	0.74-2.66	30	1.19	0.62-2.28
93	Occupations in product design, artisan craftwork, fine arts and the making of musical instruments	57	1.31	0.80-2.16	59	1.18	0.72-1.92
**S13**	**Occupations concerned with production technology**	924	0.99	0.87-1.13	928	0.87	0.76-1.00
25	Technical occupations in machine-building and automotive industry	337	0.95	0.76-1.17	339	0.91	0.73-1.13
26	Occupations in mechatronics, energy electronics and electrical engineering	295	0.96	0.76-1.20	297	0.80	0.63-1.01
27	Occupations in technical research and development, construction, and production planning and scheduling	292	1.07	0.86-1.34	292	0.93	0.74-1.16
**S14**	**Building and interior construction**	1,061	0.94	0.84-1.07	1,063	0.88	0.78-1.00
31	Occupations in construction scheduling, architecture and surveying	469	0.96	0.80-1.15	469	0.84	0.70-1.00
32	Occupations in building construction above and below ground	194	0.90	0.68-1.19	194	0.85	0.64-1.13
33	Occupations in interior construction	74	1.12	0.72-1.75	76	0.92	0.59-1.44
34	Occupations in building services engineering and technical building services	324	0.92	0.74-1.15	324	0.98	0.79-1.22
**S2**	**Personal services**	7,214	0.95	0.89-1.00	7,234	1.08	1.02-1.15
**S21**	**Food industry, gastronomy and tourism**	373	0.96	0.78-1.17	373	0.87	0.71-1.06
29	Occupations in food-production and -processing	162	1.07	0.80-1.43	162	0.76	0.56-1.04
63	Occupations in tourism, hotels and restaurants	211	0.88	0.67-1.14	211	0.97	0.74-1.25
**S22**	**Medical and non-medical health care**	3,563	0.90	0.84-0.97	3,581	1.08	1.01-1.16
81	Medical and health care occupations	3,198	0.88	0.82-0.95	3,214	1.08	1.00-1.16
82	Occupations in non-medical healthcare, body care, wellness and medical technicians	365	1.08	0.88-1.31	367	1.08	0.88-1.32
**S23**	**Service in social sector and cultural work**	3,278	1.03	0.96-1.11	3,280	1.07	0.99-1.15
83	Occupations in education and social work, housekeeping, and theology	1,543	1.03	0.93-1.14	1,547	1.09	0.99-1.20
84	Occupations in teaching and training	1,268	1.01	0.90-1.13	1,261	1.04	0.93-1.17
91	Occupations in philology, literature, humanities, social sciences, and economics	359	1.09	0.89-1.33	362	0.94	0.77-1.15
94	Occupations in the performing arts and entertainment	108	0.93	0.64-1.35	110	1.25	0.87-1.79
**S3**	**Business administration and related services**	6,809	1.04	0.98-1.10	6,839	0.97	0.92-1.03
**S31**	**Commerce and trade**	1,865	1.03	0.94-1.13	1,877	0.97	0.89-1.07
61	Occupations in purchasing, sales and trading	1,195	1.02	0.91-1.14	1,203	0.97	0.87-1.09
62	Sales occupations in retail trade	670	1.05	0.90-1.21	674	0.99	0.85-1.15
**S32**	**Business management and organisation**	980	0.92	0.81-1.04	985	0.84	0.74-0.96
71	Occupations in business management and organisation	980	0.92	0.81-1.04	985	0.84	0.74-0.96
**S33**	**Business related service occupations**	3,964	1.07	1.00-1.14	3,977	1.03	0.96-1.10
72	Occupations in financial services, accounting and tax consultancy	1,092	1.00	0.89-1.13	1,095	0.94	0.84-1.06
73	Occupations in law and public administration	2,472	1.07	0.99-1.17	2,480	1.07	0.98-1.16
92	Occupations in advertising and marketing, in commercial and editorial media design	400	1.15	0.95-1.39	402	1.05	0.87-1.26
**S4**	**Service in the IT sector and the natural sciences**	1,937	0.96	0.87-1.05	1,948	0.97	0.88-1.06
**S41**	**Service in the IT sector and the natural sciences**	1,937	0.96	0.87-1.05	1,948	0.97	0.88-1.06
41	Occupations in mathematics, biology, chemistry and physics	581	1.00	0.85-1.18	582	0.98	0.83-1.15
42	Occupations in geology, geography and environmental protection	179	1.08	0.82-1.44	181	1.05	0.79-1.39
43	Occupations in computer science, information and communication technology	1,177	0.92	0.82-1.04	1,185	0.95	0.85-1.07
**S5**	**Other commercial services**	1,193	1.07	0.95-1.20	1,198	0.98	0.87-1.11
**S51**	**Safety and security**	461	0.89	0.74-1.08	461	0.91	0.75-1.10
53	Occupations in safety and health protection, security and surveillance	369	0.94	0.76-1.16	369	0.98	0.80-1.21
01	Armed forces personnel	92	0.75	0.49-1.14	92	0.67	0.44-1.03
**S52**	**Traffic and logistics**	647	1.22	1.05-1.43	650	1.05	0.90-1.23
51	Occupations in traffic and logistics (without vehicle driving)	493	1.24	1.04-1.48	496	1.04	0.87-1.24
52	Drivers and operators of vehicles and transport equipment	154	1.15	0.85-1.56	154	1.09	0.79-1.49
**S53**	**Cleaning services**	85	0.90	0.60-1.37	87	0.90	0.60-1.36
54	Occupations in cleaning services	85	0.90	0.60-1.37	87	0.90	0.60-1.36

^a^Total effect (TE): odds ratios (OR) with 95% confidence interval (CI) derived from proportional odds models with adjustment for sex (male; female; other), age (continuously per 10 years), education (high; medium; low), employment status (permanent; fixed-term; temporary; civil servant; self-employed), weekly working hours (full time, ≥ 35h; 30-35h; 20- < 30h; < 20h).

Separate models were calculated for each occupational sector, segment, or main group, with all others excluding the specific category serving as reference group.

### Work-related psychosocial factors influencing distress

Post-pandemic (t1), occupational SARS-CoV-2 infection risk was not associated with elevated depressive and anxiety symptoms (TE, Table 4). At t0, individuals with high occupational SARS-CoV-2 infection risk exhibited increased odds of depressive and anxiety symptoms (e.g., very high risk OR=1.10, 95% CI 1.02–1.19), as the largest share of the very high occupational risk group consisted of medical health professionals (92%) whose infection risk declined sharply after the pandemic (Table 3). At t1, occupational customer contact emerged as a protective factor for depressive and anxiety symptoms, while at t0, daily customer contact was associated with more severe depressive and anxiety symptoms (TE, Table 4). However, these effects reversed after adjustment for additional occupational factors (CDE, Table S5.4 in S5 File). Models including only one additional factor at a time indicated that work-privacy conflicts and overcommitment primarily mediated the association between occupational infection risk, customer contact, and depressive and anxiety symptoms. Conversely, loneliness at work amplified risks at t0. At both time points, factors such as loneliness at work, chronic work-related stress, overcommitment to work, and high work-privacy conflicts were associated with more severe symptoms.

**Table 4:** Odds ratios for elevated depressive and anxiety symptoms by occupational risk factors.

	t1 (November 2023)			t0 (Omicron wave 2022)		
	N	OR^a^	95% CI	N	OR^a^	95% CI
Occupational SARS-CoV-2 infection risk						
Very High	3,323	0.90	0.84-0.97	3,339	1.10	1.02-1.19
High	1,507	0.99	0.89-1.09	1,510	1.06	0.96-1.18
Probable	2,482	1.04	0.95-1.13	2,497	1.08	0.99-1.17
None (reference)	12,733	1		12,782	1	
Personal customer contact						
Yes, daily	9,597	0.93	0.86-1.00	7,437	1.08	1.01-1.16
Yes, occasionally	3,826	0.94	0.86-1.02	3,441	0.99	0.91-1.07
Yes, rarely	2,980	0.94	0.85-1.03	3,630	1.06	0.98-1.15
No (reference)	3,553	1		5,495	1	
Multiple employment						
Yes – Multiple jobs	913	1.03	0.91-1.18	911	1.16	1.02-1.32
No – One job (reference)	19,132	1		19,217	1	
Loneliness at work						
Moderate/severe	1,527	3.42	3.10-3.77	4,063	2.73	2.55-2.91
None/mild (reference)	18,251	1		15,815	1	
Chronic work-related stress						
Yes (ERI > 1)	12,349	2.87	2.70-3.04	12,385	2.26	2.13-2.40
No (reference)	7,322	1		7,362	1	
Overcommitment to work [6–24]	19,946	1.30	1.29-1.31	20,028	1.21	1.20-1.22
Work-privacy conflicts						
Very high/high	2,643	2.66	2.43-2.90	4,385	2.36	2.19-2.54
Medium (reference)	5,612	1		5,627	1	
Low/very low	11,752	0.46	0.43-0.48	10,084	0.54	0.51-0.58

^a^Total effect (TE): odds ratios (OR) with 95% confidence interval (95% CI) were calculated with separate proportional odds models for each risk factor with adjustment for sex (male; female; other), age (continuously per 10 years), education (high; medium; low), employment status (permanent; fixed-term; temporary; civil servant; self-employed), weekly working hours (full time, ≥ 35h; 30-35h; 20- < 30h; < 20h).

### Stratified analyses

Elevated odds for certain occupations at t0 and t1 were observed predominantly in women (Tables S5.5 to S5.10 in S5 File). Higher occupational SARS-CoV-2 infection risk and daily customer contact at t0 increased odds in the TE models exclusively among women and elderly. Stratification by age and education showed elevated odds in medical and non-medical healthcare occupations at t0 among older individuals and those without a university degree (OR=1.21, 95% CI 1.09–1.34; OR=1.14, 95% CI 1.02–1.27, respectively). Conversely, among those with a university degree, elevated risks were observed in service occupations within the social sector and cultural work at t0 (OR=1.12, 95% CI 1.03–1.22). However, these associations did not persist in the CDE models (Table S5.10 in S5 File). In contrast, the CDE models showed high odds for younger individuals and those with lower education in IT-sector and natural sciences service roles at both time points, and in goods production, particularly manufacturing, at t1. The odds related to other occupational factors (i.e., loneliness at work, chronic work-related stress, overcommitment to work, and work-privacy conflicts) showed similar patterns across sexes, age groups, and educational levels at both time points.

## Discussion

Findings from this study revealed a stable level of psychological distress, with no notable shifts in depressive and anxiety symptoms across two time points during and after the pandemic. However, odds for more severe symptoms varied depending on employment status and type of occupation. Elevated odds were observed in occupations associated with higher SARS-CoV-2 infection exposure, particularly in the healthcare sector during the Omicron wave. After the pandemic, increased odds were especially evident in the traffic and logistics sector. Overall, associations observed tended to be weak and were influenced by psychosocial work factors, such as work-privacy conflicts and overcommitment. These individual work-related stressors, along with factors like female sex and lower educational attainment, exhibited stronger associations with severe symptoms than occupational role.

Substantial depressive and anxiety symptoms were present in 12% of the participants at both time points. Other studies among the general population observed similar prevalence proportions [33,34]. Among the working participants in DigiHero, 14.5% exhibited severe depressive symptoms (PHQ-2 ≥ 3) and 13.2% exhibited severe anxiety symptoms (GAD-2 ≥ 3) at t0, which is approximately ten percentage points lower compared to a survey conducted among non-healthcare workers in Germany during the same period [23]. These differences may result from variations in survey design, focus, period, location, or characteristics of the study population, such as age, sex, or education.

Our study focused on occupation-specific risk factors and found differences between groups. We observed, during and after the pandemic, a higher risk for more severe symptoms among job seekers compared to working individuals. This aligns with previous research linking unemployment and job insecurity to higher levels of depressive and anxiety symptoms [12,35,36]. Furthermore, part-time employment was associated with severe psychological distress, consistent with a study from Japan [36]. Conversely, we observed a protective effect for retired individuals compared to working individuals, aligning with findings from the German National Cohort during the first year of the pandemic [35], as well as among self-employed individuals. However, this contrasts with a longitudinal cohort study in Germany initiated in summer 2020 that found consistently high symptoms among freelancers and self-employed participants [37]. Another study identified a deterioration in mental health only among self-employed women who were also more affected by increased childcare responsibilities due to school and daycare closures [38]. Similarly, our study revealed a higher risk of severe depressive and anxiety symptoms among individuals experiencing high work-privacy conflicts compared to those with medium conflict, although no sex differences were observed.

During the Omicron wave, which showed the highest incidence rates of the pandemic in Germany, occupations particularly burdened by more severe depressive and anxiety symptoms included those in medical and health care occupations, with women and older individuals being especially affected. The phenomenon of higher psychological distress among nursing staff than among physicians, as described before, was replicated in our study [17]. Specifically, occupations in nursing, emergency medical services and obstetrics showed increased odds of psychological distress, as did occupations in geriatric care, and occupations in body care, in contrast to occupations in human medicine and dentistry. Furthermore, stratified analyses based on educational attainment demonstrated higher odds for psychological distress among subjects who worked in medical or non-medical healthcare professions and did not hold a university degree, compared to their counterparts with a university degree. These findings are in line with those from a large-scale U.S. survey among essential workers [18], as well as with evidence from an umbrella review focusing on healthcare workers [13]. However, these associations vanished after accounting for individual work-related psychosocial stressors, suggesting that, unlike academic medical professions, the elevated risk observed among nursing staff is mainly driven by these stressors.

After the pandemic, elevated risks were observed in the traffic and logistics sector (including warehouse, postal, logistics clerks, and passenger service staff), particularly among women and older workers. A Korean survey among 353 logistics workers found that these employees experience high time pressure, physical labor, and job insecurity contributing to presenteeism which increases the risk of mental health problems [39]. This may explain the higher psychological distress observed in this group in DigiHero, though it does not clarify why these effects occurred only after the pandemic.

Other recognized occupational risk factors for more severe symptoms identified in this study include overcommitment to work and chronic work-related stress. These factors were documented in an umbrella review of prospective cohort studies on depressive disorders [40] and in studies of workers during the pandemic [22,23,41]. Loneliness at work emerged as a major risk factor for depressive and anxiety symptoms within this study population, potentially constituting the strongest occupational risk factor at both time points. Similar findings were reported in a 2023 German quota sample, where individuals experiencing loneliness were significantly more likely to report depressive and anxiety symptoms [42]. Notably, the psychological distress of loneliness remained consistent across sexes, age, and educational attainment, matching prior findings of minimal variation by these factors [42]. A survey conducted 2020 in Japan found low co-worker and supervisor support strongly linked to loneliness [43], highlighting their key role in preventing related mental health issues.

### Strengths and limitations

The large convenience sample represents a strength, as it enabled a detailed examination of factors for psychological distress across specific occupational groups, rather than relying solely on generalized occupational categories. Furthermore, validated scales were used to assess symptoms and occupational stressors at two time points. However, the findings should be interpreted with caution due to limitations. First, the lack of representativeness inherent to convenience sampling, along with self-selection, limits the generalizability of the findings to the general population. Participants with better health and digital literacy appear to have participated more often in DigiHero. Compared to both, the 2022 German census [44] and the non-respondents of this survey, the study sample included a higher proportion of women (60% versus 51% and 53%, respectively) and more individuals holding a university degree (64% versus 20% and 37%). The study population was also selective in respect to occupational composition, with health and legal professions being overrepresented while occupations in cleaning or transport logistics were underrepresented. Consequently, findings primarily reflect the study population and should be interpreted cautiously, with limited generalizability to less healthy, less digitally literate, or underrepresented occupational groups. Furthermore, overall participation in DigiHero was low, ranging from 2% to 8% across regions. Second, data from t0 were collected retrospectively, which may introduce recall bias, a common limitation to retrospective studies. This could have influenced how participants recalled symptoms during the early 2022 Omicron wave, potentially leading to under- or over-reporting of PHQ-4 symptoms. Third, although it became evident that infection risks for workers in different occupations varied over time [6], the classification of occupations according to SARS-CoV-2 infection risk was based on considerations from the beginning of the pandemic [22,26]. This approach was chosen to provide a consistent reference point, facilitating comparability across studies. Furthermore, occupational differences in COVID-19 outcomes became less pronounced over time [6], Consequently, occupational infection risks appear to have been less differentiated in early 2022 and late 2023 compared with the early stages of the pandemic. To account for this convergence of occupational risks, sensitivity analyses were conducted in which the very high and high occupational SARS-CoV-2 risk categories were combined. These analyses similarly indicated a decline in the impact of occupational SARS-CoV-2 risk on severe symptoms from t0 (OR=1.09, 95% CI 1.02–1.16) to t1 (OR=0.93, 95% CI 0.87–0.99).

## Conclusions

The impact of occupational SARS-CoV-2 infection risk on psychological distress may be transient as it vanished after the pandemic. However, 13% of working participants experienced substantial symptoms of depression and anxiety post-pandemic, indicating that workers’ mental health remains an important public health concern particularly in light of additional emerging crises such as the war in Ukraine [45]. Unlike pandemic-related burdens, established occupational risk factors (e.g., chronic work-related stress, work-privacy conflicts) remain stable across time and pandemic conditions and were also more pronounced at both time points. Our findings highlight the need for sustained efforts to promote workers’ mental well-being and reduce the occupational risks that contribute to depression and related disorders, particularly in the groups represented in this study. Since these psychosocial work factors are modifiable, there is considerable potential for prevention.

## Supporting information

S1 File STROBE checklist. (PDF)

S2 File Study variables, including psychometric instruments and occupational and demographic characteristics. (PDF)

S3 File Directed Acyclic Graph illustrating the effect of occupational SARS-CoV-2 infection risk, personal customer contact, and other work-related stressors on depressive and anxiety symptoms assessed with the brief 4-item Patient Health Questionnaire-4. (PDF)

S4 File Adjusted odds ratios and 95% confidence intervals for increased depression and anxiety symptoms among working, job seeking and non-working participants. (PDF)

S5 File Additional proportional odds model analyses for elevated depressive and anxiety symptoms among working participants. (PDF)
