# Supplementary material for: Occupational risk factors for depression and anxiety symptoms: Insights from a large cohort study during and after the SARS-CoV-2 pandemic
Source: PLoS One. 2026 Apr 15;21(4):e0346871. doi: 10.1371/journal.pone.0346871 (PMC13082607; doi:10.1371/journal.pone.0346871)
Supplement: S2 File — (PDF) [file pone.0346871.s002.pdf]

Article: Occupational risk factors for depression and anxiety symptoms: Insights from a large cohort study during and after the SARS-CoV-2 pandemic (Casjens et al.)

S2 File. Study variables, including psychometric instruments and occupational and demographic characteristics

Type	Variable / Domain	Instrument / Values	Survey	Reference
Psychometric	Depression and anxiety	Four-item Patient Health Questionnaire (PHQ-4)	t1, t0	27
Occupational	Professional activity	Working; Job seeker; Not working	t1	
Occupational	Employment status	Permanent; Fixed-term; Temporary; Civil servant; Self-employed	t1	
Occupational	Number of jobs	One job; Multiple jobs	t1	
Occupational	Weekly working hours	Continuous	t1, t0	
Occupational	Occupation	German Classification of Occupations coding scheme	t1, t0	24
Occupational	Occupational SARS-CoV-2 infection risk	None; Probable; High; Very high	t1	22
Psychometric	Work-privacy conflicts	Copenhagen Psychosocial Questionnaire item: “To what extent do the demands of your work interfere with your private and family life?”	t1, t0	28
Psychometric	Loneliness at work	Three-item UCLA Loneliness Scale (adapted)	t1, t0	29, 30
Psychometric	Chronic work-related stress	Ten-item effort-reward imbalance questionnaire	t1	31
Psychometric	Over-commitment to work	Six-item over-commitment questionnaire	t1	32
Demographic	Age	Continuous, in years	t0, t1	
Demographic	Sex	Male; Female; Other	t0	
Demographic	Education	Low: ≤10 years of schooling; Medium: >10 years of schooling; High: university degree	t0	
