# Supplementary material for: Occupational risk factors for depression and anxiety symptoms: Insights from a large cohort study during and after the SARS-CoV-2 pandemic
Source: PLoS One. 2026 Apr 15;21(4):e0346871. doi: 10.1371/journal.pone.0346871 (PMC13082607; doi:10.1371/journal.pone.0346871)
Supplement: S3 File — (PDF) [file pone.0346871.s003.pdf]

Article: Occupational risk factors for depression and anxiety symptoms: Insights from a large cohort study during and after the SARS-CoV-2 pandemic **(Casjens et al.)**

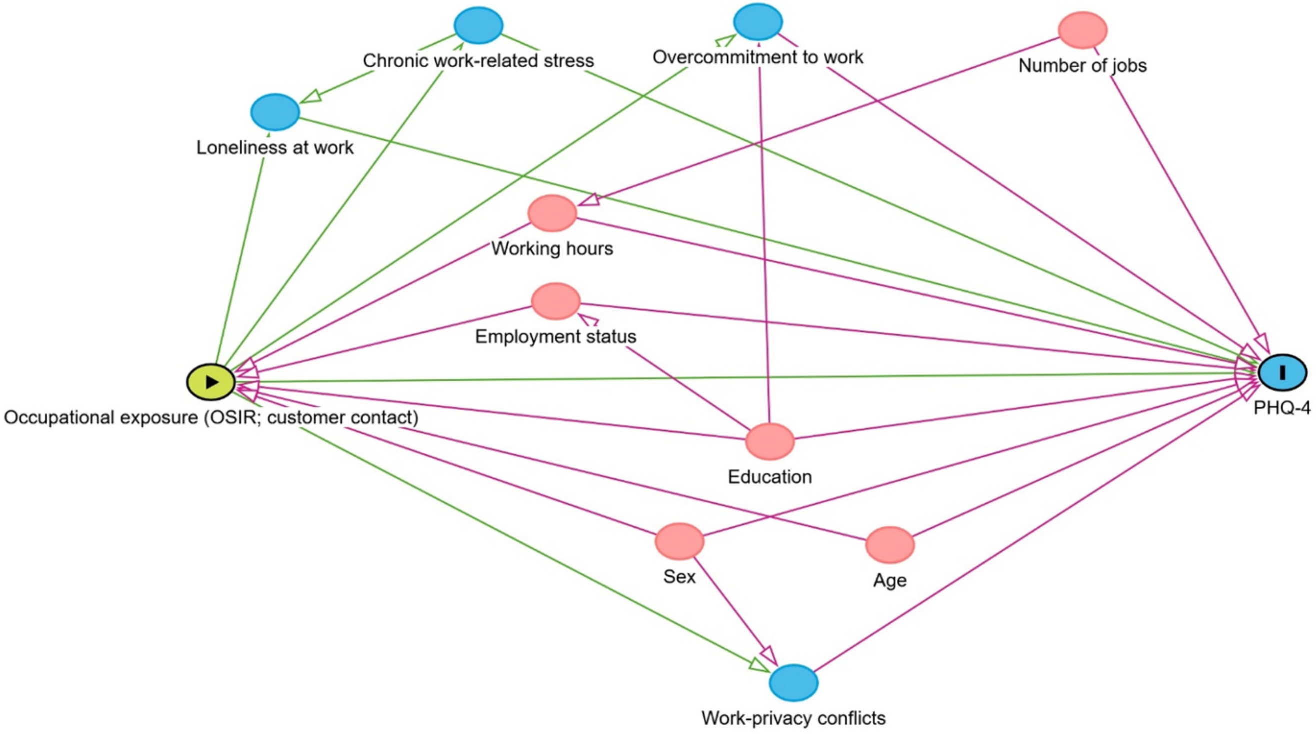

S3 File. Directed Acyclic Graph (DAG) illustrating the effect of occupational SARS-CoV-2 infection risk (OSIR), personal customer contact, and other work-related stressors on depressive and anxiety symptoms assessed with the brief 4-item Patient Health Questionnaire-4 (PHQ-4)
