# Supplementary material for: Occupational risk factors for depression and anxiety symptoms: Insights from a large cohort study during and after the SARS-CoV-2 pandemic
Source: PLoS One. 2026 Apr 15;21(4):e0346871. doi: 10.1371/journal.pone.0346871 (PMC13082607; doi:10.1371/journal.pone.0346871)
Supplement: S4 File — (PDF) [file pone.0346871.s004.pdf]

**Article:** Occupational risk factors for depression and anxiety symptoms: Insights from a large cohort study during and after the SARS-CoV-2 pandemic **(Casjens et al.)**

**S4 File**. Adjusted odds ratios and 95% confidence intervals for increased depression and anxiety symptoms among working, job seeking and non-working participants (n=33,771 participants)

| | | t1 (November 2023) | | t0 (Omicron wave 2022) | |
|---|---|---|---|---|---|
| | N | OR | 95% CI | OR | 95% CI |
| Professional activity | | | | | |
| Job seeker | 470 | 2.11 | 1.78-2.50 | 1.54 | 1.29-1.83 |
| Not working | 12,663 | 1.06 | 1.00-1.11 | 0.78 | 0.74-0.82 |
| Working | 20,638 | 1 | | 1 | |
| Educational attainment | | | | | |
| Low: ≤10y schooling | 444 | 1.88 | 1.57-2.25 | 1.70 | 1.41-2.04 |
| Medium: >10y schooling | 11,589 | 1.23 | 1.18-1.29 | 1.17 | 1.12-1.23 |
| High: University degree | 21,738 | 1 | | 1 | |
| Sex | | | | | |
| Female | 20,345 | 1.66 | 1.59-1.74 | 1.95 | 1.86-2.04 |
| Other | 39 | 5.08 | 2.81-9.19 | 5.45 | 3.02-9.83 |
| Male | 13,387 | 1 | | 1 | |
| Age [per 10 years] | 33,771 | 0.83 | 0.82-0.85 | 0.80 | 0.79-0.82 |

Odds ratios (OR) and 95% confidence intervals (95% CI) derived from a proportional odds model at t0 and t1, respectively.
