# Supplementary material for: Occupational risk factors for depression and anxiety symptoms: Insights from a large cohort study during and after the SARS-CoV-2 pandemic
Source: PLoS One. 2026 Apr 15;21(4):e0346871. doi: 10.1371/journal.pone.0346871 (PMC13082607; doi:10.1371/journal.pone.0346871)
Supplement: S5 File — (PDF) [file pone.0346871.s005.pdf]

Article: Occupational risk factors for depression and anxiety symptoms: Insights from a large cohort study during and after the SARS-CoV-2 pandemic **(Casjens et al.)**

S5 File. Additional proportional odds model analyses for elevated depressive and anxiety symptoms among working participants

Table S5.1. Univariable estimates for the adjustment set for elevated depression and anxiety symptoms among working participants

	t1 (November 2023)			t0 (Omicron wave 2022)		
	N	OR	95% CI	N	OR	95% CI
Sex						
Female	13,434	1.54	1.46-1.63	13,434	1.85	1.75-1.96
Other	21	10.11	4.74-21.57	21	7.15	3.34-15.30
Male	7,274	1		7,274	1	
Age [per 10 years]	20,697	0.88	0.86-0.90	20,697	0.85	0.83-0.87
Educational attainment						
Low: ≤10y schooling	199	2.09	1.61-2.72	199	2.00	1.54-2.61
Medium: >10y schooling	7,426	1.22	1.15-1.28	7,426	1.22	1.15-1.29
High: University degree	13,123	1		13,123	1	
Employment status						
Fixed-term	847	1.33	1.16-1.51	847	1.24	1.09-1.41
Temporary	36	1.77	0.98-3.19	36	1.96	1.09-3.53
Civil servant	2,062	0.99	0.91-1.08	2,062	1.01	0.93-1.10
Self-employed	1,499	0.67	0.60-0.74	1,499	0.78	0.70-0.86
Permanent	16,369	1		16,369	1	
Weekly working hours						
<20h	979	1.07	0.94-1.20	1,034	1.28	1.14-1.44
20-30h	2,184	1.22	1.12-1.33	2,205	1.38	1.27-1.50
30-35h	2,866	1.20	1.11-1.30	2,702	1.31	1.22-1.42
Full time, ≥35h	14,438	1		14,619	1	

Odds ratios (OR) and 95% confidence intervals (95% CI) derived from separate proportional odds models.

Article: Occupational risk factors for depression and anxiety symptoms: Insights from a large cohort study during and after the SARS-CoV-2 pandemic **(Casjens et al.)**

Table S5.2. Odds ratios for depression and anxiety by occupational sector, segment and main group, controlled direct effects

KldB	Occupations	t1 (November 2023)			t0 (Omicron wave 2022)		
		N	OR*	95% CI	N	OR*	95% CI
**S1**	**Production of goods**	2,772	1.09	1.00-1.19	2,788	0.98	0.90-1.07
**S11**	**Agriculture, forestry and horticulture**	284	1.13	0.89-1.43	287	1.18	0.93-1.48
11	Occupations in agriculture, forestry, and farming	208	0.99	0.75-1.30	210	1.09	0.83-1.42
12	Occupations in gardening and floristry	76	1.64	1.05-2.57	77	1.43	0.94-2.20
**S12**	**Manufacturing**	576	1.21	1.02-1.43	583	1.12	0.94-1.32
21	Occupations in production and processing of raw materials, glass- and ceramic-making and -processing	54	0.73	0.41-1.30	54	0.95	0.55-1.65
22	Occupations in plastic-making and -processing, and wood-working and -processing	123	0.94	0.66-1.35	123	0.78	0.54-1.13
23	Occupations in paper-making and -processing, printing, and in technical media design	52	1.62	0.93-2.82	52	2.13	1.25-3.62
24	Occupations in metal-making and -working, and in metal construction	268	1.29	1.01-1.64	274	1.13	0.88-1.44
28	Occupations in textile- and leather-making and -processing	28	1.78	0.89-3.56	27	1.45	0.71-2.98
93	Occupations in product design, artisan craftwork, fine arts and the making of musical instruments	51	1.48	0.86-2.55	53	1.24	0.73-2.11
**S13**	**Occupations concerned with production technology**	898	1.08	0.94-1.25	903	0.91	0.79-1.05
25	Technical occupations in machine-building and automotive industry	327	1.09	0.86-1.37	328	1.01	0.81-1.28
26	Occupations in mechatronics, energy electronics and electrical engineering	285	0.99	0.77-1.27	289	0.79	0.62-1.02
27	Occupations in technical research and development, construction, and production planning and scheduling	286	1.15	0.91-1.45	286	0.94	0.74-1.19
**S14**	**Building and interior construction**	1,014	0.98	0.86-1.12	1,015	0.92	0.81-1.05
31	Occupations in construction scheduling, architecture and surveying	440	1.13	0.93-1.37	442	0.97	0.80-1.18
32	Occupations in building construction above and below ground	191	0.84	0.62-1.15	191	0.83	0.61-1.12
33	Occupations in interior construction	70	0.92	0.57-1.51	71	0.90	0.56-1.45
34	Occupations in building services engineering and technical building services	313	0.90	0.71-1.14	311	0.93	0.74-1.18
**S2**	**Personal services**	6,946	0.72	0.67-0.77	6,974	0.87	0.81-0.92
**S21**	**Food industry, gastronomy and tourism**	353	0.91	0.74-1.13	355	0.90	0.72-1.11
29	Occupations in food-production and -processing	153	1.08	0.78-1.48	154	0.80	0.58-1.11
63	Occupations in tourism, hotels and restaurants	200	0.80	0.60-1.07	201	0.98	0.74-1.29
**S22**	**Medical and non-medical health care**	3,431	0.74	0.69-0.80	3,452	0.91	0.84-0.98
81	Medical and health care occupations	3,091	0.71	0.65-0.77	3,111	0.90	0.83-0.98
82	Occupations in non-medical healthcare, body care, wellness and medical technicians	340	1.17	0.94-1.45	341	1.01	0.81-1.25
**S23**	**Service in social sector and cultural work**	3,162	0.83	0.76-0.90	3,167	0.90	0.83-0.97
83	Occupations in education and social work, housekeeping, and theology	1,492	0.95	0.85-1.06	1,493	1.01	0.91-1.12
84	Occupations in teaching and training	1,231	0.68	0.60-0.77	1,232	0.77	0.68-0.87
91	Occupations in philology, literature, humanities, social sciences, and economics	339	1.04	0.83-1.30	341	0.90	0.72-1.12

KldB	Occupations	t1 (November 2023)			t0 (Omicron wave 2022)		
		N	OR*	95% CI	N	OR*	95% CI
94	Occupations in the performing arts and entertainment	100	0.98	0.66-1.47	101	1.42	0.96-2.10
**S3**	**Business administration and related services**	6,573	1.19	1.12-1.27	6,610	1.11	1.04-1.17
**S31**	**Commerce and trade**	1,793	1.02	0.92-1.12	1,802	1.00	0.91-1.11
61	Occupations in purchasing, sales and trading	1,147	1.07	0.95-1.21	1,152	1.04	0.92-1.17
62	Sales occupations in retail trade	646	0.93	0.79-1.09	650	0.94	0.80-1.10
**S32**	**Business management and organisation**	941	1.02	0.89-1.17	946	0.91	0.80-1.04
71	Occupations in business management and organisation	941	1.02	0.89-1.17	946	0.91	0.80-1.04
**S33**	**Business related service occupations**	3,839	1.27	1.18-1.36	3,862	1.18	1.10-1.27
72	Occupations in financial services, accounting and tax consultancy	1,052	1.05	0.93-1.19	1,059	1.01	0.89-1.15
73	Occupations in law and public administration	2,412	1.32	1.21-1.45	2,425	1.23	1.13-1.34
92	Occupations in advertising and marketing, in commercial and editorial media design	375	1.29	1.05-1.58	378	1.22	1.00-1.49
**S4**	**Service in the IT sector and the natural sciences**	1,887	1.14	1.03-1.27	1,895	1.08	0.98-1.20
**S41**	**Service in the IT sector and the natural sciences**	1,887	1.14	1.03-1.27	1,895	1.08	0.98-1.20
41	Occupations in mathematics, biology, chemistry and physics	564	1.12	0.94-1.32	567	1.03	0.87-1.22
42	Occupations in geology, geography and environmental protection	175	1.18	0.87-1.60	174	1.13	0.83-1.53
43	Occupations in computer science, information and communication technology	1,148	1.13	0.99-1.29	1,154	1.10	0.96-1.24
**S5**	**Other commercial services**	1,151	1.17	1.03-1.33	1,157	1.06	0.93-1.20
**S51**	**Safety and security**	451	1.00	0.82-1.23	453	0.98	0.80-1.19
53	Occupations in safety and health protection, security and surveillance	362	1.02	0.82-1.28	364	1.05	0.85-1.30
1	Armed forces personnel	89	0.91	0.57-1.43	89	0.72	0.46-1.14
**S52**	**Traffic and logistics**	620	1.29	1.09-1.52	623	1.11	0.95-1.31
51	Occupations in traffic and logistics (without vehicle driving)	474	1.35	1.12-1.63	477	1.12	0.93-1.35
52	Drivers and operators of vehicles and transport equipment	146	1.10	0.78-1.53	146	1.10	0.78-1.54
**S53**	**Cleaning services**	80	1.17	0.75-1.82	81	1.02	0.66-1.59
54	Occupations in cleaning services	80	1.17	0.75-1.82	81	1.02	0.66-1.59

*Controlled direct effect (CDE): odds ratios (OR) with 95% confidence interval (CI) derived from proportional odds models with adjustment for sex (male; female; other), age (continuously per 10 years), education (high; medium; low), employment relationship (permanent; fixed-term; temporary; civil servant; self-employed), weekly working hours (full time, ≥35h; 30-35h; 20-<30h; <20h), loneliness at work (none/mild; moderate/severe); chronic work-related stress (no; yes); overcommitment to work; work-privacy conflicts (very high/high; medium; low/very low).

Separate models were calculated for each occupation, with all others excluding the specific category serving as reference group.

KldB, Occupational groups according to the German Classification of Occupations

Table S5.3. Modeling the risk of elevated depressive and anxiety symptoms (PHQ-4 categories) among 20,948 working participants by occupational group. No analyses were conducted for occupations with fewer than 30 individuals.

KldB-3	Occupations	Total effects*						Controlled direct effects†					
		t1 (November 2023)			t0 (Omicron wave 2022)			t1 (November 2023)			t0 (Omicron wave 2022)		
		N	OR	95% CI	N	OR	95% CI	N	OR	95% CI	N	OR	95% CI
111	Occupations in farming	117	0.97	0.68-1.38	118	1.18	0.84-1.66	113	0.99	0.68-1.44	113	1.23	0.86-1.76
112	Occupations in animal husbandry	<30	-	-	<30	-	-	<30	-	-	<30	-	-
113	Occupations in horsekeeping	<30	-	-	<30	-	-	<30	-	-	<30	-	-
114	Occupations in fishing	<30	-	-	<30	-	-	<30	-	-	<30	-	-
115	Occupations in animal care	<30	-	-	<30	-	-	<30	-	-	<30	-	-
116	Occupations in vini- and viticulture	<30	-	-	<30	-	-	<30	-	-	<30	-	-
117	Occupations in forestry, hunting and landscape preservation	39	1.03	0.56-1.87	40	0.79	0.43-1.46	39	0.89	0.46-1.71	39	0.71	0.37-1.37
121	Occupations in gardening	76	1.41	0.91-2.16	76	1.28	0.85-1.93	69	1.68	1.05-2.69	69	1.62	1.03-2.53
122	Occupations in floristry	<30	-	-	<30	-	-	<30	-	-	<30	-	-
211	Occupations in underground and surface mining and blasting engineering	<30	-	-	<30	-	-	<30	-	-	<30	-	-
212	Conditioning and processing of natural stone and minerals, production of building materials	<30	-	-	<30	-	-	<30	-	-	<30	-	-
213	Occupations in industrial glass-making and -processing	<30	-	-	<30	-	-	<30	-	-	<30	-	-
214	Occupations in industrial ceramic-making and -processing	<30	-	-	<30	-	-	<30	-	-	<30	-	-
221	Occupations in plastic- and rubber-making and -processing	92	1.14	0.78-1.68	92	1.00	0.67-1.49	88	1.07	0.70-1.61	88	0.85	0.55-1.29
222	Occupations in colour coating and varnishing	<30	-	-	<30	-	-	<30	-	-	<30	-	-
223	Occupations in wood-working and -processing	33	0.60	0.30-1.22	33	0.62	0.30-1.31	30	0.70	0.32-1.57	30	0.62	0.27-1.45
231	Technical occupations in paper-making and -processing and packaging	<30	-	-	<30	-	-	<30	-	-	<30	-	-
232	Occupations in technical media design	<30	-	-	<30	-	-	<30	-	-	<30	-	-
233	Occupations in photography and photographic technology	<30	-	-	<30	-	-	<30	-	-	<30	-	-
234	Occupations in printing technology, print finishing, and book binding	<30	-	-	<30	-	-	<30	-	-	<30	-	-
241	Occupations in metal-making	<30	-	-	<30	-	-	<30	-	-	<30	-	-
242	Occupations in metalworking	139	1.27	0.92-1.74	142	1.07	0.78-1.47	130	1.27	0.90-1.80	133	1.06	0.75-1.50
243	Occupations in treatment of metal surfaces	<30	-	-	<30	-	-	<30	-	-	<30	-	-
244	Occupations in metal constructing and welding	71	1.06	0.68-1.65	71	1.00	0.63-1.59	66	1.01	0.62-1.66	68	0.98	0.59-1.61
245	Occupations in precision mechanics and tool making	30	1.31	0.67-2.57	30	1.37	0.70-2.71	30	1.78	0.86-3.69	30	1.69	0.83-3.46

KldB-3	Occupations	Total effects*						Controlled direct effects†					
		t1 (November 2023)			t0 (Omicron wave 2022)			t1 (November 2023)			t0 (Omicron wave 2022)		
		N	OR	95% CI	N	OR	95% CI	N	OR	95% CI	N	OR	95% CI
251	Occupations in machine-building and -operating	154	1.00	0.73-1.37	156	0.96	0.70-1.32	149	1.18	0.84-1.66	150	1.21	0.87-1.69
252	Technical occupations in the automotive, aeronautic, aerospace and ship building industries	183	0.91	0.68-1.21	183	0.87	0.65-1.16	178	1.02	0.74-1.39	178	0.88	0.64-1.19
261	Occupations in mechatronics, automation and control technology	87	0.79	0.51-1.21	87	0.56	0.36-0.88	83	0.81	0.50-1.31	84	0.56	0.35-0.92
262	Technical occupations in energy technologies	67	1.00	0.63-1.60	67	0.76	0.47-1.24	66	1.13	0.69-1.86	67	0.73	0.43-1.22
263	Occupations in electrical engineering	141	1.05	0.76-1.44	143	1.01	0.73-1.40	136	1.04	0.73-1.46	138	1.01	0.71-1.43
271	Occupations in technical research and development	160	1.23	0.92-1.65	160	1.03	0.77-1.39	158	1.23	0.91-1.68	158	1.00	0.73-1.36
272	Draftspersons, technical designers, and model makers	31	0.63	0.30-1.30	31	0.51	0.24-1.08	31	0.77	0.36-1.65	31	0.66	0.31-1.44
273	Technical occupations in production planning and scheduling	101	1.01	0.69-1.47	101	0.93	0.63-1.36	97	1.14	0.76-1.71	97	0.93	0.61-1.42
281	Occupations in textile making	<30	-	-	<30	-	-	<30	-	-	<30	-	-
282	Occupations in the production of clothing and other textile products	<30	-	-	<30	-	-	<30	-	-	<30	-	-
283	Occupations in leather- and fur-making and -processing	<30	-	-	<30	-	-	<30	-	-	<30	-	-
291	Occupations in beverage production	<30	-	-	<30	-	-	<30	-	-	<30	-	-
292	Occupations in the production of foodstuffs, confectionery and tobacco products	119	1.03	0.73-1.46	120	0.79	0.55-1.12	113	1.02	0.71-1.48	115	0.79	0.54-1.14
293	Cooking occupations	<30	-	-	<30	-	-	<30	-	-	<30	-	-
311	Occupations in construction scheduling and supervision, and architecture	398	0.97	0.80-1.17	398	0.81	0.66-0.98	372	1.03	0.84-1.28	375	0.88	0.71-1.09
312	Occupations in surveying and cartography	71	0.92	0.58-1.45	71	1.01	0.65-1.55	68	1.84	1.13-3.00	67	1.64	1.03-2.60
321	Occupations in building construction	84	0.96	0.63-1.47	84	0.97	0.64-1.49	83	1.00	0.64-1.58	83	1.03	0.66-1.61
322	Occupations in civil engineering	110	0.85	0.59-1.24	110	0.76	0.52-1.12	108	0.74	0.49-1.11	108	0.70	0.46-1.05
331	Floor layers	<30	-	-	<30	-	-	<30	-	-	<30	-	-
332	Painters and varnishers, plasterers, occupations in the waterproofing of buildings, preservation of structures and wooden building components	<30	-	-	<30	-	-	<30	-	-	<30	-	-
333	Occupations in the interior construction and dry walling, insulation, carpentry, glazing, roller shutter and jalousie installation	44	0.98	0.55-1.73	46	0.96	0.54-1.69	41	0.69	0.36-1.34	43	0.95	0.51-1.77
341	Occupations in building services engineering	212	0.93	0.71-1.23	213	0.98	0.75-1.28	207	1.01	0.76-1.35	206	1.02	0.77-1.36
342	Occupations in plumping, sanitation, heating, ventilating, and air conditioning	77	1.03	0.67-1.59	76	1.00	0.65-1.55	71	0.73	0.45-1.19	70	0.67	0.41-1.10
343	Occupations in building services and waste disposal	35	0.63	0.31-1.28	35	0.96	0.49-1.88	35	0.72	0.34-1.52	35	1.10	0.55-2.18
411	Occupations in mathematics and statistics	41	1.44	0.78-2.63	41	1.07	0.59-1.95	39	1.23	0.65-2.33	39	1.06	0.56-2.03
412	Occupations in biology	193	1.03	0.78-1.36	192	1.10	0.84-1.43	188	1.10	0.82-1.47	188	1.10	0.83-1.46

KldB-3	Occupations	Total effects*						Controlled direct effects†					
		t1 (November 2023)			t0 (Omicron wave 2022)			t1 (November 2023)			t0 (Omicron wave 2022)		
		N	OR	95% CI	N	OR	95% CI	N	OR	95% CI	N	OR	95% CI
413	Occupations in chemistry	218	1.03	0.80-1.33	220	1.04	0.81-1.35	211	1.21	0.92-1.59	214	1.16	0.89-1.51
414	Occupations in physics	129	0.81	0.57-1.14	129	0.70	0.49-1.00	126	0.94	0.65-1.36	126	0.73	0.50-1.06
421	Occupations in geology, geography and meteorology	67	1.57	1.00-2.45	68	1.44	0.92-2.25	66	1.75	1.09-2.82	66	1.65	1.03-2.65
422	Occupations in environmental protection engineering	<30	-	-	<30	-	-	<30	-	-	<30	-	-
423	Occupations in environmental protection management and environmental protection consulting	83	0.93	0.61-1.42	84	0.97	0.63-1.49	80	1.03	0.65-1.64	79	1.11	0.71-1.75
431	Occupations in computer science	116	0.68	0.47-0.99	118	0.73	0.50-1.04	111	0.80	0.53-1.19	113	0.76	0.51-1.13
432	Occupations in IT-system-analysis, IT-application-consulting and IT-sales	216	0.94	0.72-1.22	216	0.90	0.69-1.17	212	1.03	0.77-1.37	211	1.00	0.76-1.33
433	Occupations in IT-network engineering, IT-coordination, IT-administration and IT-organisation	382	0.92	0.75-1.13	384	0.96	0.78-1.17	375	1.02	0.82-1.27	379	0.98	0.79-1.22
434	Occupations in software development and programming	463	1.00	0.83-1.20	467	1.06	0.89-1.28	450	1.38	1.13-1.69	451	1.35	1.11-1.64
511	Technical occupations in railway, aircraft and ship operation	90	0.81	0.53-1.23	89	0.80	0.52-1.22	85	0.80	0.51-1.28	85	0.91	0.58-1.43
512	Occupations in the inspection and maintenance of traffic infrastructure	49	1.00	0.58-1.74	49	0.63	0.35-1.14	48	1.46	0.81-2.61	48	0.67	0.36-1.26
513	Occupations in warehousing and logistics, in postal and other delivery services, and in cargo handling	141	1.52	1.11-2.09	141	1.03	0.74-1.42	135	1.68	1.20-2.35	134	1.13	0.80-1.59
514	Service occupations in passenger traffic	<30	-	-	<30	-	-	<30	-	-	<30	-	-
515	Occupations in traffic surveillance and control	33	1.24	0.66-2.34	33	1.07	0.55-2.07	33	1.14	0.57-2.28	33	1.05	0.52-2.12
516	Management assistants in transport and logistics	153	1.32	0.97-1.79	156	1.36	1.01-1.83	146	1.40	1.01-1.94	149	1.40	1.03-1.92
521	Driver of vehicles in road traffic	107	1.16	0.81-1.66	108	1.26	0.87-1.84	101	1.18	0.79-1.77	102	1.35	0.90-2.01
522	Drivers of vehicles in railway traffic	<30	-	-	<30	-	-	<30	-	-	<30	-	-
523	Aircraft pilots	<30	-	-	<30	-	-	<30	-	-	<30	-	-
524	Ship's officers and masters	<30	-	-	<30	-	-	<30	-	-	<30	-	-
525	Drivers and operators of construction and transportation vehicles and equipment	<30	-	-	<30	-	-	<30	-	-	<30	-	-
531	Occupations in physical security, personal protection, fire protection and workplace safety	117	1.13	0.80-1.61	118	1.17	0.82-1.67	114	1.00	0.69-1.46	116	1.02	0.70-1.47
532	Occupations in police and criminal investigation, jurisdiction and the penal institution	234	0.83	0.64-1.08	233	0.91	0.70-1.18	230	0.99	0.75-1.32	230	1.10	0.84-1.44
533	Occupations in occupational health and safety administration, public health authority, and disinfection	<30	-	-	<30	-	-	<30	-	-	<30	-	-
541	Occupations in cleaning services	85	0.90	0.60-1.37	87	0.90	0.60-1.36	80	1.17	0.75-1.82	81	1.02	0.66-1.59

KldB-3	Occupations	Total effects*						Controlled direct effects†					
		t1 (November 2023)			t0 (Omicron wave 2022)			t1 (November 2023)			t0 (Omicron wave 2022)		
		N	OR	95% CI	N	OR	95% CI	N	OR	95% CI	N	OR	95% CI
611	Occupations in purchasing and sales	781	0.98	0.85-1.13	786	0.95	0.82-1.09	756	1.00	0.86-1.16	761	1.00	0.86-1.16
612	Trading occupations	343	1.07	0.87-1.31	345	1.03	0.84-1.26	321	1.16	0.93-1.45	320	1.14	0.91-1.43
613	Occupations in real estate and facility management	71	1.21	0.78-1.89	72	0.95	0.61-1.47	70	1.39	0.87-2.22	71	1.03	0.65-1.63
621	Sales occupations in retail trade (without product specialisation)	287	1.09	0.87-1.36	289	1.00	0.80-1.25	277	0.97	0.76-1.24	278	0.94	0.75-1.20
622	Sales occupations (retail trade) selling clothing, electronic devices, furniture, motor vehicles and other durables	111	1.03	0.72-1.48	110	1.10	0.77-1.57	107	1.07	0.72-1.58	107	1.18	0.81-1.72
623	Sales occupations (retail) selling foodstuffs	177	1.24	0.94-1.65	178	1.15	0.86-1.52	172	0.84	0.62-1.14	174	0.94	0.70-1.26
624	Sales occupations (retail) selling drugstore products, pharmaceuticals, medical supplies and healthcare goods	80	0.64	0.41-0.99	81	0.58	0.37-0.90	76	0.73	0.46-1.17	76	0.61	0.38-0.98
625	Sales occupations (retail) selling books, art, antiques, musical instruments, recordings or sheet music	<30	-	-	<30	-	-	<30	-	-	<30	-	-
631	Occupations in tourism and the sports (and fitness) industry	56	0.72	0.43-1.21	58	0.85	0.51-1.40	54	1.03	0.60-1.79	57	1.15	0.68-1.93
632	Occupations in hotels	51	0.73	0.42-1.26	51	0.85	0.51-1.42	47	0.51	0.27-0.94	47	0.72	0.40-1.27
633	Gastronomy occupations	78	1.12	0.73-1.73	77	1.18	0.77-1.81	74	0.94	0.59-1.49	73	1.10	0.70-1.72
634	Occupations in event organisation and management	<30	-	-	<30	-	-	<30	-	-	<30	-	-
711	Managing directors and executive board members	133	0.72	0.51-1.02	134	0.59	0.41-0.85	126	0.64	0.44-0.95	127	0.57	0.39-0.84
712	Legislators and senior officials of special interest organisations	31	1.07	0.54-2.11	32	0.75	0.37-1.50	31	0.91	0.45-1.85	31	0.63	0.30-1.33
713	Occupations in business organisation and strategy	344	0.88	0.71-1.09	343	0.77	0.62-0.96	332	0.96	0.76-1.20	333	0.81	0.64-1.02
714	Office clerks and secretaries	313	0.95	0.77-1.17	315	0.92	0.74-1.13	299	1.13	0.90-1.42	301	1.09	0.87-1.36
715	Occupations in human resources management and personnel service	159	1.15	0.85-1.56	161	1.18	0.88-1.59	153	1.34	0.98-1.85	154	1.22	0.90-1.66
721	Occupations in insurance and financial services	349	0.90	0.73-1.10	350	0.86	0.70-1.06	334	1.09	0.87-1.36	338	1.01	0.82-1.26
722	Occupations in accounting, controlling and auditing	522	1.04	0.88-1.22	523	0.93	0.79-1.10	502	1.13	0.95-1.35	505	1.01	0.85-1.21
723	Occupations in tax consultancy	221	1.11	0.86-1.42	222	1.11	0.86-1.43	216	0.84	0.64-1.10	216	1.01	0.77-1.32
731	Occupations in legal services, jurisdiction, and other officers of the court	364	1.14	0.93-1.39	362	0.94	0.77-1.15	355	1.25	1.01-1.54	353	1.02	0.82-1.26
732	Occupations in public administration	2,024	1.06	0.97-1.16	2,035	1.08	0.99-1.18	1,974	1.32	1.20-1.45	1,990	1.23	1.12-1.36
733	Occupations in media, documentation and information services	84	0.88	0.58-1.34	83	1.20	0.81-1.78	83	1.12	0.72-1.74	82	1.65	1.09-2.48
811	Doctors' receptionists and assistants	412	1.08	0.90-1.31	413	1.03	0.85-1.24	395	1.06	0.86-1.29	395	0.99	0.81-1.20
812	Laboratory occupations in medicine	131	1.22	0.88-1.70	132	1.10	0.81-1.51	127	1.34	0.95-1.89	128	1.11	0.79-1.55

KldB-3	Occupations	Total effects*						Controlled direct effects†					
		t1 (November 2023)			t0 (Omicron wave 2022)			t1 (November 2023)			t0 (Omicron wave 2022)		
		N	OR	95% CI	N	OR	95% CI	N	OR	95% CI	N	OR	95% CI
813	Occupations in nursing, emergency medical services and obstetrics	1,271	0.96	0.86-1.07	1,273	1.36	1.22-1.51	1,228	0.64	0.57-0.72	1,236	0.94	0.84-1.06
814	Occupations in human medicine and dentistry	551	0.82	0.70-0.98	556	0.79	0.67-0.93	535	0.66	0.55-0.79	538	0.70	0.59-0.84
815	Occupations in veterinary medicine and non-medical animal health practitioners	40	0.82	0.46-1.49	40	0.78	0.43-1.40	40	0.65	0.34-1.24	39	0.71	0.38-1.34
816	Occupations in psychology and non-medical psychotherapy	142	0.53	0.38-0.75	144	0.65	0.47-0.90	137	0.68	0.46-0.99	141	0.79	0.56-1.12
817	Occupations in non-medical therapy and alternative medicine	385	0.76	0.62-0.93	389	0.93	0.77-1.13	370	0.85	0.68-1.05	375	0.99	0.80-1.21
818	Occupations in pharmacy	266	0.87	0.69-1.10	267	1.12	0.89-1.41	259	0.84	0.65-1.08	259	1.14	0.89-1.45
821	Occupations in geriatric care	81	1.20	0.80-1.81	81	1.64	1.08-2.51	75	0.97	0.62-1.51	74	1.06	0.68-1.67
822	Occupations providing nutritional advice or health counselling, and occupations in wellness	99	0.85	0.57-1.25	99	0.71	0.48-1.05	93	1.11	0.73-1.70	92	0.78	0.51-1.19
823	Occupations in body care	49	1.12	0.66-1.91	50	1.79	1.03-3.12	42	1.19	0.63-2.23	43	1.58	0.86-2.91
824	Occupations in funeral services	<30	-	-	<30	-	-	<30	-	-	<30	-	-
825	Technical occupations in medicine, orthopaedic and rehabilitation	128	1.17	0.84-1.63	128	0.96	0.68-1.33	122	1.35	0.94-1.92	123	1.01	0.70-1.44
831	Occupations in education and social work, and pedagogic specialists in social care work	1,446	1.01	0.91-1.13	1,450	1.06	0.96-1.18	1,401	0.94	0.84-1.05	1,404	0.98	0.88-1.09
832	Occupations in housekeeping and consumer counselling	65	1.26	0.79-2.01	65	1.46	0.92-2.30	59	1.03	0.61-1.74	58	1.53	0.93-2.53
833	Occupations in theology and church community work	32	1.38	0.73-2.61	32	1.47	0.81-2.66	32	1.26	0.64-2.46	31	1.26	0.66-2.41
841	Teachers in schools of general education	733	1.06	0.91-1.23	728	1.10	0.95-1.28	714	0.60	0.51-0.71	713	0.71	0.60-0.83
842	Teachers for occupation-specific subjects at vocational schools and in-company instructors in vocational training	207	0.99	0.76-1.29	204	0.81	0.62-1.06	199	0.82	0.62-1.10	198	0.68	0.51-0.91
843	Teachers and researchers at universities and colleges	144	0.91	0.66-1.25	144	0.86	0.62-1.18	141	0.92	0.66-1.29	141	0.82	0.58-1.15
844	Teachers at educational institutions other than schools (except driving, flying and sports instructors)	169	0.98	0.73-1.31	170	1.31	0.99-1.75	162	0.86	0.62-1.18	165	1.28	0.95-1.73
845	Driving, flying and sports instructors at educational institutions other than schools	<30	-	-	<30	-	-	<30	-	-	<30	-	-
911	Occupations in philology	97	1.19	0.80-1.76	99	0.93	0.63-1.36	91	1.09	0.72-1.66	92	0.80	0.53-1.23
912	Occupations in the humanities	124	1.19	0.85-1.67	123	1.11	0.79-1.55	116	1.10	0.76-1.58	115	1.12	0.78-1.60
913	Occupations in the social sciences	86	0.98	0.65-1.46	87	0.86	0.57-1.30	83	0.82	0.53-1.28	84	0.75	0.48-1.17

KldB-3	Occupations	Total effects*						Controlled direct effects†					
		t1 (November 2023)			t0 (Omicron wave 2022)			t1 (November 2023)			t0 (Omicron wave 2022)		
		N	OR	95% CI	N	OR	95% CI	N	OR	95% CI	N	OR	95% CI
914	Occupations in economics	52	0.87	0.51-1.49	53	0.75	0.44-1.29	49	1.25	0.70-2.23	50	0.92	0.52-1.64
921	Occupations in advertising and marketing	165	0.92	0.68-1.23	167	1.05	0.79-1.41	154	1.04	0.75-1.43	155	1.28	0.94-1.75
922	Occupations in public relations	72	1.46	0.95-2.25	72	0.99	0.64-1.53	67	1.49	0.93-2.39	68	1.08	0.69-1.71
923	Occupations in publishing and media management	52	0.89	0.53-1.49	52	0.96	0.57-1.62	50	1.12	0.64-1.95	50	1.08	0.62-1.87
924	Occupations in editorial work and journalism	111	1.54	1.07-2.21	111	1.11	0.77-1.60	104	1.70	1.16-2.49	105	1.28	0.87-1.88
931	Occupations in product and industrial design	<30	-	-	<30	-	-	<30	-	-	<30	-	-
932	Occupations in interior design, visual marketing, and interior decoration	<30	-	-	<30	-	-	<30	-	-	<30	-	-
933	Occupations in artisan craftwork and fine arts	31	1.36	0.71-2.61	31	1.05	0.53-2.10	<30	-	-	<30	-	-
934	Artisans designing ceramics and glassware	<30	-	-	<30	-	-	<30	-	-	<30	-	-
935	Artisans working with metal	<30	-	-	<30	-	-	<30	-	-	<30	-	-
936	Occupations in musical instrument making	<30	-	-	<30	-	-	<30	-	-	<30	-	-
941	Musicians, singers and conductors	42	0.81	0.44-1.47	44	1.76	1.00-3.10	40	0.88	0.47-1.68	40	2.03	1.13-3.67
942	Actors, dancers, athletes and related occupations	<30	-	-	<30	-	-	<30	-	-	<30	-	-
943	Presenters and entertainers	<30	-	-	<30	-	-	<30	-	-	<30	-	-
944	Occupations in theatre, film and television productions	<30	-	-	<30	-	-	<30	-	-	<30	-	-
945	Occupations in event technology, cinematography, and sound engineering	<30	-	-	<30	-	-	<30	-	-	<30	-	-
946	Occupations in stage, costume and prop design,	<30	-	-	<30	-	-	<30	-	-	<30	-	-
947	Technical and management occupations in museums and exhibitions	<30	-	-	<30	-	-	<30	-	-	<30	-	-
011	Commissioned officers	34	0.41	0.19-0.88	34	0.53	0.25-1.09	34	0.41	0.18-0.96	34	0.56	0.26-1.21
012	Senior non-commissioned officers and higher	30	1.02	0.50-2.07	30	0.73	0.35-1.54	30	1.05	0.50-2.20	30	0.68	0.32-1.45
013	Junior non-commissioned officers	<30	-	-	<30	-	-	<30	-	-	<30	-	-
014	Armed forces personnel in other ranks	<30	-	-	<30	-	-	<30	-	-	<30	-	-

*Total effects (TE): odds ratios (OR) with 95% confidence interval (CI) derived from proportional odds models with adjustment for sex (male; female; other), age (continuously per 10 years), education (high; medium; low), employment status (permanent; fixed-term; temporary; civil servant; self-employed), weekly working hours (full time, ≥35h; 30-35h; 20-<30h; <20h).

†Controlled direct effects (CDE): ORs with 95% CI derived from proportional odds models with adjustment for sex (male; female; other), age (continuously per 10 years), education (high; medium; low), employment relationship (permanent; fixed-term; temporary; civil servant; self-employed), weekly working hours (full time, ≥35h; 30-35h; 20-<30h; <20h), loneliness at work (none/mild; moderate/severe); chronic work-related stress (no; yes); overcommitment to work; work-privacy conflicts (very high/high; medium; low/very low).

Separate models were calculated for each occupation, with all others excluding the specific category serving as reference group.

KldB-3, Occupational groups according to the German Classification of Occupations

Article: Occupational risk factors for depression and anxiety symptoms: Insights from a large cohort study during and after the SARS-CoV-2 pandemic **(Casjens et al.)**

Table S5.4. Odds ratios for depression and anxiety by occupational SARS-CoV-2 infection risk and customer contact, controlled direct effects

	t1 (November 2023)		t0 (Omicron wave 2022)			t1 (November 2023)		t0 (Omicron wave 2022)	
	N	OR* (95% CI)	N	OR* (95% CI)		N	OR* (95% CI)	N	OR* (95% CI)
Occupational SARS-CoV-2 infection risk					Personal customer contact				
Very High	3,209	0.74 (0.68-0.80)	3,227	0.92 (0.85-1.00)	Yes, daily	9,250	0.61 (0.56-0.66)	7,183	0.82 (0.76-0.88)
High	1,448	0.88 (0.79-0.99)	1,451	1.01 (0.91-1.13)	Yes, occasionally	3,691	0.75 (0.68-0.83)	3,306	0.85 (0.78-0.93)
Probable	2,419	1.22 (1.11-1.33)	2,440	1.18 (1.08-1.29)	Yes, rarely	2,881	0.85 (0.77-0.94)	3,516	0.93 (0.86-1.02)
None (ref.)	12,253	1	12,306	1	No (ref.)	3,433	1	5,310	1
Loneliness at work									
None/mild (ref.)	17,840	1	15,448	1		17,774	1	15,361	1
Moderate/severe	1,489	2.59 (2.34-2.87)	3,976	2.27 (2.11-2.43)		1,481	2.43 (2.19-2.69)	3,954	2.22 (2.07-2.38)
Chronic work-related stress									
No (ref.)	7,184	1	7,219	1		7,159	1	7,169	1
Yes (ERI > 1)	12,145	1.26 (1.17-1.35)	12,205	1.09 (1.02-1.17)		12,096	1.28 (1.20-1.38)	12,146	1.10 (1.02-1.17)
Overcommitment to work [6-24]	19,329	1.24 (1.23-1.25)	19,424	1.16 (1.15-1.17)		19,255	1.25 (1.23-1.26)	19,315	1.16 (1.15-1.17)
Work-privacy conflicts									
Very high/high	2,572	1.80 (1.64-1.98)	4,253	1.88 (1.74-2.04)		2,551	1.82 (1.66-2.00)	4,232	1.90 (1.75-2.05)
Medium (ref.)	5,420	1	5,444	1		5,401	1	5,418	1
Low/very low	11,337	0.77 (0.71-0.82)	9,727	0.72 (0.67-0.77)		11,303	0.76 (0.71-0.82)	9,665	0.71 (0.66-0.76)

*Controlled direct effects (CDE): Odds ratios (OR) with 95% confidence interval (CI) derived from proportional odds models with additional adjustment for sex (male; female; other), age (continuously per 10 years), education (high; medium; low), employment relationship (permanent; fixed-term; temporary; civil servant; self-employed), weekly working hours (full time, ≥35h; 30-35h; 20-<30h; <20h).

Table S5.5. Odds ratios for increased depression and anxiety symptoms by occupational classification and occupational risk factors with **stratification by sex**, total effects

		t1 (November 2023)			t0 (Omicron wave 2022)			t1 (November 2023)			t0 (Omicron wave 2022)		
		N	OR	95% CI	N	OR	95% CI	N	OR	95% CI	N	OR	95% CI
		Men (n=7,274)						Women (n=13,343)					
**Occupations**													
**S1**	**Production of goods**	1,936	0.99	0.89-1.10	1,942	0.93	0.84-1.04	952	1.08	0.95-1.22	963	0.93	0.83-1.06
S11	Agriculture, forestry and horticulture	137	1.03	0.74-1.43	139	0.91	0.64-1.27	160	1.22	0.90-1.63	163	1.30	0.98-1.71
S12	Manufacturing	414	1.03	0.84-1.25	416	1.08	0.88-1.31	194	1.38	1.07-1.80	198	1.05	0.81-1.37
S13	Occupations concerned with production technology	720	1.00	0.85-1.16	722	0.91	0.78-1.07	203	0.98	0.75-1.27	205	0.75	0.58-0.98
S14	Building and interior construction	665	0.95	0.81-1.12	665	0.91	0.78-1.07	395	0.94	0.78-1.14	397	0.86	0.71-1.04
**S2**	**Personal services**	1,347	0.96	0.85-1.08	1,359	1.04	0.92-1.17	5,863	0.94	0.88-1.00	5,870	1.09	1.02-1.17
S21	Food industry, gastronomy and tourism	137	1.16	0.84-1.61	136	0.90	0.64-1.27	235	0.85	0.66-1.10	236	0.86	0.68-1.10
S22	Medical and non-medical health care	588	0.96	0.81-1.14	594	1.00	0.84-1.18	2,974	0.89	0.82-0.97	2,986	1.10	1.02-1.19
S23	Service in social sector and cultural work	622	0.92	0.78-1.09	629	1.11	0.94-1.31	2,654	1.05	0.96-1.14	2,648	1.05	0.97-1.14
**S3**	**Business administration and related services**	1,866	1.08	0.97-1.20	1,876	1.05	0.95-1.17	4,937	1.03	0.96-1.10	4,957	0.95	0.89-1.02
S31	Commerce and trade	639	1.14	0.97-1.34	644	1.00	0.85-1.18	1,223	0.99	0.89-1.11	1,230	0.97	0.87-1.09
S32	Business management and organisation	301	0.87	0.68-1.10	301	0.89	0.70-1.12	678	0.94	0.82-1.09	683	0.83	0.71-0.96
S33	Business related service occupations	926	1.09	0.95-1.26	931	1.14	0.99-1.32	3,036	1.06	0.98-1.15	3,044	1.00	0.92-1.08
**S4**	**Service in the IT sector and the natural sciences**	1,176	0.93	0.81-1.05	1,183	0.97	0.86-1.10	757	0.99	0.86-1.14	761	0.95	0.82-1.09
S41	Service in the IT sector and the natural sciences	1,176	0.93	0.81-1.05	1,183	0.97	0.86-1.10	757	0.99	0.86-1.14	761	0.95	0.82-1.09
**S5**	**Other commercial services**	711	1.05	0.90-1.23	708	1.02	0.87-1.20	481	1.10	0.92-1.31	489	0.95	0.80-1.13
S51	Safety and security	294	0.99	0.77-1.27	292	0.94	0.72-1.21	166	0.80	0.59-1.09	168	0.89	0.66-1.19
S52	Traffic and logistics	391	1.14	0.93-1.39	390	1.09	0.88-1.34	256	1.34	1.06-1.70	260	1.01	0.80-1.28
S53	Cleaning services	<30	-	-	<30	-	-	59	1.08	0.66-1.77	61	0.90	0.56-1.45
**Other occupational risk factors**													
	Occupational SARS-CoV-2 infection risk												
	Very High	548	0.97	0.82-1.16	554	1.01	0.85-1.20	2,773	0.89	0.82-0.97	2,783	1.13	1.04-1.23
	High	395	1.04	0.84-1.28	396	1.07	0.87-1.32	1,112	0.96	0.85-1.09	1,114	1.06	0.94-1.20
	Probable	729	0.99	0.84-1.16	733	1.03	0.88-1.21	1,753	1.06	0.96-1.18	1,764	1.10	1.00-1.22
	None (ref.)	5,364	1		5,385	1		7,352	1		7,379	1	
	Customer contact												
	Yes, daily	2,700	0.91	0.80-1.04	1,956	0.99	0.87-1.12	6,892	0.94	0.85-1.03	5,474	1.12	1.04-1.22
	Yes, occasionally	1,629	0.95	0.82-1.09	1,346	0.93	0.80-1.06	2,193	0.94	0.84-1.05	2,092	1.03	0.93-1.14
	Yes, rarely	1,302	0.96	0.83-1.12	1,627	1.07	0.94-1.21	1,672	0.92	0.81-1.03	1,999	1.04	0.94-1.16
	No (ref.)	1,370	1		2,086	1		2,179	1		3,403	1	
	Multiple employment												
	Yes - Multiple jobs	272	1.00	0.78-1.28	274	1.13	0.89-1.45	639	1.04	0.89-1.21	635	1.14	0.98-1.34
	No - One job (ref.)	6,764	1		6,794	1		12,351	1		12,405	1	
	Loneliness at work												
	Moderate/severe	557	3.89	3.30-4.59	1,248	2.97	2.63-3.34	967	3.17	2.81-3.58	2,807	2.63	2.43-2.85
	None/mild (ref.)	6,397	1		5,728	1		11,838	1		10,075	1	
	Chronic work-related stress												
	Yes (ERI > 1)	3,904	3.04	2.75-3.37	3,919	2.39	2.16-2.64	8,435	2.79	2.59-3.01	8,455	2.21	2.05-2.37
	No (ref.)	3,007	1		3,022	1		4,306	1		4,331	1	
	Overcommitment to work	7,009	1.32	1.30-1.34	7,041	1.24	1.22-1.26	12,918	1.29	1.27-1.30	12,967	1.20	1.19-1.21
	Work-privacy conflicts												
	Very high/high	848	2.68	2.29-3.13	1,267	2.29	2.00-2.63	1,787	2.66	2.39-2.96	3,112	2.39	2.18-2.62
	Medium (ref)	1,934	1		2,001	1		3,676	1		3,621	1	
	Low/very low	4,250	0.49	0.44-0.54	3,796	0.57	0.51-0.64	7,493	0.44	0.41-0.48	6,280	0.53	0.49-0.57

*Total effects (TE): odds ratios (OR) with 95% confidence interval (CI) derived from proportional odds models with adjustment for sex (male; female; other), age (continuously per 10 years), education (high; medium; low), employment status (permanent; fixed-term; temporary; civil servant; self-employed), weekly working hours (full time, ≥35h; 30-35h; 20-<30h; <20h).

Separate models were calculated for each occupation, with all others excluding the specific category serving as reference group.

Table S5.6. Odds ratios for increased depression and anxiety symptoms by occupational classification and occupational risk factors with **stratification by age**, total effects

		t1 (November 2023)			t0 (Omicron wave 2022)			t1 (November 2023)			t0 (Omicron wave 2022)		
		N	OR	95% CI	N	OR	95% CI	N	OR	95% CI	N	OR	95% CI
		Young people (≤50 years at t1, n=9,988)						Old people (>50 years at t1, n=10,709)					
**Occupations**													
**S1**	**Production of goods**	1,305	1.00	0.89-1.12	1,312	0.92	0.82-1.03	1,587	1.05	0.94-1.17	1,597	0.95	0.85-1.06
S11	Agriculture, forestry and horticulture	118	0.93	0.66-1.32	119	0.98	0.70-1.38	179	1.29	0.98-1.72	183	1.25	0.95-1.65
S12	Manufacturing	293	1.17	0.94-1.46	296	1.04	0.83-1.30	317	1.14	0.92-1.42	320	1.11	0.89-1.38
S13	Occupations concerned with production technology	478	0.98	0.82-1.17	480	0.85	0.70-1.01	446	1.01	0.83-1.23	448	0.92	0.75-1.12
S14	Building and interior construction	416	0.93	0.77-1.13	417	0.93	0.77-1.13	645	0.95	0.81-1.11	646	0.84	0.72-0.99
**S2**	**Personal services**	3,665	0.91	0.84-0.98	3,664	1.02	0.94-1.11	3,549	0.99	0.91-1.08	3,570	1.15	1.06-1.25
S21	Food industry, gastronomy and tourism	201	0.89	0.68-1.17	200	0.87	0.67-1.14	172	1.02	0.76-1.37	173	0.85	0.63-1.14
S22	Medical and non-medical health care	1,865	0.81	0.73-0.89	1,874	0.98	0.89-1.08	1,698	1.02	0.92-1.13	1,707	1.21	1.09-1.34
S23	Service in social sector and cultural work	1,599	1.10	0.99-1.23	1,590	1.09	0.98-1.21	1,679	0.97	0.87-1.08	1,690	1.05	0.94-1.16
**S3**	**Business administration and related services**	3,151	1.09	1.01-1.19	3,158	1.01	0.93-1.10	3,658	0.98	0.90-1.06	3,681	0.93	0.85-1.00
S31	Commerce and trade	764	1.10	0.95-1.26	768	1.01	0.88-1.16	1,101	0.97	0.86-1.10	1,109	0.94	0.83-1.06
S32	Business management and organisation	438	0.99	0.82-1.19	439	0.92	0.77-1.11	542	0.86	0.72-1.02	546	0.78	0.65-0.92
S33	Business related service occupations	1,949	1.09	0.99-1.20	1,951	1.03	0.94-1.14	2,015	1.04	0.94-1.14	2,026	1.01	0.92-1.11
**S4**	**Service in the IT-sector and the natural sciences**	1,126	1.07	0.94-1.21	1,133	1.09	0.96-1.23	811	0.84	0.73-0.98	815	0.84	0.73-0.98
S41	Service in the IT-sector and the natural sciences	1,126	1.07	0.94-1.21	1,133	1.09	0.96-1.23	811	0.84	0.73-0.98	815	0.84	0.73-0.98
**S5**	**Other commercial services**	519	0.91	0.76-1.09	520	0.88	0.74-1.05	674	1.23	1.06-1.44	678	1.11	0.95-1.30
S51	Safety and security	247	0.81	0.62-1.05	248	0.84	0.65-1.09	214	1.04	0.79-1.37	213	1.04	0.79-1.37
S52	Traffic and logistics	241	1.05	0.82-1.34	241	0.90	0.71-1.16	406	1.36	1.12-1.65	409	1.18	0.97-1.44
S53	Cleaning services	31	0.79	0.40-1.55	31	1.00	0.52-1.92	54	0.99	0.58-1.69	56	0.85	0.51-1.44
**Other occupational risk factors**													
	Occupational SARS-CoV-2 infection risk												
	Very High	1,749	0.79	0.71-0.88	1,757	0.98	0.88-1.08	1,574	1.05	0.94-1.17	1,582	1.26	1.13-1.40
	High	720	0.93	0.80-1.08	720	0.98	0.84-1.13	787	1.04	0.90-1.20	790	1.15	1.00-1.33
	Probable	1,200	0.96	0.85-1.09	1,203	1.00	0.89-1.13	1,282	1.10	0.98-1.24	1,294	1.13	1.01-1.27
	None (ref.)	6,097	1		6,107	1		6,636	1		6,675	1	
	Customer contact												
	Yes, daily	4,467	0.89	0.80-0.98	3,626	1.03	0.94-1.13	5,130	0.97	0.86-1.08	3,811	1.14	1.03-1.26
	Yes, occasionally	1,776	0.90	0.80-1.02	1,466	0.96	0.85-1.08	2,050	0.97	0.86-1.11	1,975	1.00	0.89-1.13
	Yes, rarely	1,528	0.91	0.80-1.03	1,645	1.05	0.94-1.18	1,452	0.97	0.84-1.11	1,985	1.06	0.94-1.19
	No (ref.)	1,956	1		2,999	1		1,597	1		2,496	1	
	Multiple employment												
	Yes - Multiple jobs	465	1.04	0.87-1.24	464	1.23	1.03-1.47	448	1.05	0.87-1.27	447	1.09	0.90-1.32
	No - One job (ref.)	9,301	1		9,323	1		9,831	1		9,894	1	
	Loneliness at work												
	Moderate/severe	729	3.54	3.08-4.08	1,994	2.66	2.42-2.92	798	3.29	2.87-3.76	2,069	2.79	2.54-3.06
	None/mild (ref.)	9,001	1		7,750	1		9,250	1		8,065	1	
	Chronic work-related stress												
	Yes (ERI > 1)	6,135	2.69	2.47-2.92	6,148	2.07	1.91-2.25	6,214	3.03	2.78-3.30	6,237	2.44	2.25-2.66
	No (ref.)	3,494	1		3,499	1		3,828	1		3,863	1	
	Overcommitment to work	9,723	1.28	1.26-1.29	9,745	1.20	1.19-1.21	10,223	1.31	1.30-1.33	10,283	1.21	1.20-1.23
	Work-privacy conflicts									-			-
	Very high/high	1,330	2.73	2.42-3.09	2,260	2.50	2.25-2.78	1,313	2.57	2.26-2.91	2,125	2.22	1.99-2.47
	Medium (ref)	2,855	1		2,776	1		2,757	1		2,851	1	
	Low/very low	5,566	0.49	0.45-0.54	4,737	0.62	0.56-0.67	6,186	0.42	0.38-0.46	5,347	0.48	0.44-0.53

*Total effects (TE): odds ratios (OR) with 95% confidence interval (CI) derived from proportional odds models with adjustment for sex (male; female; other), age (continuously per 10 years), education (high; medium; low), employment status (permanent; fixed-term; temporary; civil servant; self-employed), weekly working hours (full time, ≥35h; 30-35h; 20-<30h; <20h).

Separate models were calculated for each occupation, with all others excluding the specific category serving as reference group.

Table S5.7. Odds ratios for increased depression and anxiety symptoms by occupational classification and occupational risk factors with **stratification by education**, total effects

		t1 (November 2023)			t0 (Omicron wave 2022)			t1 (November 2023)			t0 (Omicron wave 2022)		
		N	OR	95% CI	N	OR	95% CI	N	OR	95% CI	N	OR	95% CI
		Low education (no university degree, n=7,625)						High education (university degree, n=13,123)					
**Occupations**													
**S1**	**Production of goods**	1,081	1.08	0.94-1.23	1,090	0.94	0.83-1.08	1,811	0.99	0.89-1.10	1,819	0.92	0.83-1.02
S11	Agriculture, forestry and horticulture	117	1.31	0.93-1.86	118	1.25	0.89-1.76	180	1.03	0.77-1.36	184	1.05	0.79-1.38
S12	Manufacturing	320	1.17	0.95-1.45	323	1.06	0.85-1.31	290	1.10	0.88-1.39	293	1.08	0.86-1.36
S13	Occupations concerned with production technology	294	0.97	0.77-1.22	297	0.77	0.61-0.97	630	1.00	0.85-1.17	631	0.93	0.79-1.09
S14	Building and interior construction	350	0.96	0.78-1.19	352	0.93	0.75-1.15	711	0.93	0.80-1.09	711	0.86	0.74-1.00
**S2**	**Personal services**	2,329	0.91	0.82-1.00	2,331	1.03	0.94-1.14	4,885	0.96	0.89-1.03	4,903	1.11	1.03-1.19
S21	Food industry, gastronomy and tourism	222	0.91	0.70-1.18	221	0.81	0.63-1.05	151	1.03	0.76-1.41	152	0.96	0.70-1.30
S22	Medical and non-medical health care	1,552	0.90	0.81-1.01	1,557	1.14	1.02-1.27	2,011	0.90	0.81-0.99	2,024	1.04	0.94-1.14
S23	Service in social sector and cultural work	555	0.99	0.84-1.18	553	0.89	0.76-1.06	2,723	1.03	0.95-1.12	2,727	1.12	1.03-1.22
**S3**	**Business administration and related services**	2,877	0.99	0.90-1.08	2,888	0.96	0.88-1.05	3,932	1.09	1.01-1.17	3,951	0.99	0.92-1.06
S31	Commerce and trade	1,026	0.96	0.85-1.09	1,031	0.97	0.85-1.10	839	1.13	0.98-1.29	846	0.98	0.86-1.13
S32	Business management and organisation	331	0.79	0.64-0.98	334	0.80	0.65-0.99	649	1.00	0.86-1.17	651	0.87	0.75-1.02
S33	Business related service occupations	1,520	1.07	0.96-1.20	1,523	1.03	0.92-1.15	2,444	1.07	0.98-1.17	2,454	1.03	0.95-1.13
**S4**	**Service in the IT sector and the natural sciences**	401	1.19	0.98-1.45	404	1.12	0.92-1.35	1,536	0.90	0.81-1.00	1,544	0.93	0.84-1.03
S41	Service in the IT sector and the natural sciences	401	1.19	0.98-1.45	404	1.12	0.92-1.35	1,536	0.90	0.81-1.00	1,544	0.93	0.84-1.03
**S5**	**Other commercial services**	703	1.07	0.91-1.25	706	1.04	0.89-1.22	490	1.07	0.90-1.29	492	0.92	0.77-1.10
S51	Safety and security	209	0.89	0.66-1.19	209	1.05	0.78-1.41	252	0.92	0.72-1.19	252	0.82	0.64-1.07
S52	Traffic and logistics	425	1.19	0.98-1.43	426	1.06	0.88-1.29	222	1.25	0.97-1.62	224	1.03	0.79-1.33
S53	Cleaning services	69	0.88	0.55-1.40	71	0.91	0.58-1.42	16			16		
**Other occupational risk factors**													
	Occupational SARS-CoV-2 infection risk												
	Very High	1,454	0.92	0.81-1.03	1,458	1.15	1.03-1.30	1,869	0.89	0.80-0.98	1,881	1.06	0.96-1.17
	High	727	0.95	0.82-1.11	731	0.99	0.85-1.15	780	1.02	0.88-1.17	779	1.14	0.99-1.31
	Probable	1,009	1.00	0.87-1.14	1,013	1.11	0.97-1.27	1,473	1.08	0.97-1.20	1,484	1.06	0.95-1.18
	None (ref.)	4,201	1		4,217	1		8,532	1		8,565	1	
	Customer contact												
	Yes, daily	3,744	0.95	0.85-1.06	3,136	1.03	0.92-1.14	5,853	0.91	0.82-1.00	4,301	1.11	1.02-1.22
	Yes, occasionally	1,097	1.03	0.89-1.19	1,024	0.97	0.84-1.12	2,729	0.89	0.80-1.00	2,417	1.00	0.90-1.11
	Yes, rarely	1,001	1.00	0.86-1.17	1,156	0.95	0.83-1.09	1,979	0.90	0.80-1.02	2,474	1.12	1.01-1.24
	No (ref.)	1,524	1		2,055	1		2,029	1		3,440	1	
	Multiple employment												
	Yes - Multiple jobs	372	0.95	0.78-1.17	372	1.19	0.97-1.46	541	1.09	0.92-1.30	539	1.12	0.95-1.33
	No - One job (ref.)	7,019	1		7,047	1		12,113	1		12,170	1	
	Loneliness at work												
	Moderate/severe	531	3.34	2.83-3.93	1,344	2.69	2.41-3.01	996	3.49	3.09-3.94	2,719	2.76	2.54-2.99
	None/mild (ref.)	6,755	1		5,972	1		11,496	1		9,843	1	
	Chronic work-related stress												
	Yes (ERI > 1)	4,710	2.88	2.61-3.18	4,720	2.31	2.10-2.54	7,639	2.86	2.65-3.08	7,665	2.24	2.08-2.41
	No (ref.)	2,535	1		2,548	1		4,790	1		4,814	1	
	Overcommitment to work	7,349	1.29	1.28-1.31	7,377	1.21	1.20-1.23	12,597	1.30	1.29-1.31	12,651	1.21	1.20-1.22
	Work-privacy conflicts												
	Very high/high	995	2.41	2.09-2.78	1,751	2.46	2.17-2.78	1,648	2.83	2.53-3.17	2,634	2.31	2.09-2.54
	Medium (ref)	1,984	1		1,974	1		3,628	1		3,653	1	
	Low/very low	4,399	0.46	0.41-0.50	3,677	0.56	0.51-0.63	7,353	0.46	0.42-0.49	6,407	0.53	0.49-0.58

*Total effects (TE): odds ratios (OR) with 95% confidence interval (CI) derived from proportional odds models with adjustment for sex (male; female; other), age (continuously per 10 years), education (high; medium; low), employment status (permanent; fixed-term; temporary; civil servant; self-employed), weekly working hours (full time, ≥35h; 30-35h; 20-<30h; <20h).

Separate models were calculated for each occupation, with all others excluding the specific category serving as reference group.

Table S5.8. Odds ratios for increased depression and anxiety symptoms by occupational classification and occupational risk factors with **stratification by sex**, controlled direct effects

		t1 (November 2023)			t0 (Omicron wave 2022)			t1 (November 2023)			t0 (Omicron wave 2022)		
		N	OR	95% CI	N	OR	95% CI	N	OR	95% CI	N	OR	95% CI
		Men (n=7,274)						Women (n=13,343)					
**Occupations**													
**S1**	**Production of goods**	1,867	0.98	0.87-1.10	1871	0.93	0.83-1.04	901	1.29	1.13-1.48	913	1.07	0.93-1.22
S11	Agriculture, forestry and horticulture	133	0.97	0.68-1.38	133	0.82	0.57-1.18	151	1.27	0.92-1.75	154	1.46	1.08-1.98
S12	Manufacturing	390	1.05	0.84-1.30	394	1.16	0.94-1.43	184	1.56	1.18-2.06	187	1.06	0.80-1.40
S13	Occupations concerned with production technology	705	1.06	0.90-1.25	707	0.90	0.77-1.07	192	1.22	0.92-1.62	195	0.94	0.71-1.24
S14	Building and interior construction	639	0.87	0.73-1.04	637	0.90	0.75-1.07	374	1.17	0.95-1.44	377	0.98	0.80-1.20
**S2**	**Personal services**	1,305	0.83	0.73-0.95	1,311	0.89	0.78-1.02	5,637	0.68	0.63-0.74	5,659	0.85	0.79-0.91
S21	Food industry, gastronomy and tourism	130	1.13	0.79-1.62	130	0.86	0.59-1.24	222	0.81	0.62-1.06	225	0.91	0.71-1.18
S22	Medical and non-medical health care	571	0.83	0.69-1.00	573	0.82	0.68-0.99	2,859	0.73	0.67-0.79	2878	0.92	0.84-1.00
S23	Service in social sector and cultural work	604	0.81	0.67-0.98	608	1.01	0.85-1.21	2,556	0.82	0.75-0.90	2,556	0.87	0.79-0.95
**S3**	**Business administration and related services**	1,808	1.10	0.98-1.23	1,818	1.09	0.97-1.22	4,759	1.24	1.15-1.34	4,786	1.13	1.05-1.21
S31	Commerce and trade	622	1.04	0.87-1.24	625	0.92	0.77-1.10	1168	1.01	0.89-1.14	1,174	1.05	0.93-1.18
S32	Business management and organisation	291	0.79	0.61-1.02	290	0.86	0.67-1.12	649	1.13	0.96-1.32	655	0.93	0.80-1.09
S33	Business related service occupations	895	1.24	1.07-1.45	903	1.29	1.11-1.50	2,942	1.28	1.18-1.39	2,957	1.16	1.07-1.27
**S4**	**Service in the IT sector and the natural sciences**	1,148	1.10	0.95-1.27	1,152	1.10	0.96-1.26	735	1.20	1.03-1.40	739	1.05	0.91-1.22
S41	Service in the IT sector and the natural sciences	1,148	1.10	0.95-1.27	1,152	1.10	0.96-1.26	735	1.20	1.03-1.40	739	1.05	0.91-1.22
**S5**	**Other commercial services**	686	1.02	0.86-1.22	683	1.03	0.87-1.23	464	1.38	1.14-1.66	473	1.09	0.91-1.31
S51	Safety and security	288	0.97	0.74-1.27	287	0.91	0.70-1.20	162	1.04	0.75-1.44	165	1.06	0.78-1.43
S52	Traffic and logistics	374	1.07	0.86-1.34	373	1.10	0.88-1.38	246	1.64	1.27-2.11	250	1.16	0.90-1.48
S53	Cleaning services	24	0.82	0.35-1.92	23	1.27	0.54-2.95	56	1.32	0.78-2.22	58	0.92	0.56-1.54
**Other occupational risk factors**													
	Occupational SARS-CoV-2 infection risk												
	Very High	532	0.83	0.69-1.01	534	0.83	0.68-1.01	2,675	0.72	0.66-0.79	2,691	0.94	0.86-1.03
	High	379	1.01	0.81-1.26	376	1.10	0.88-1.38	1,069	0.84	0.73-0.95	1,075	0.99	0.87-1.12
	Probable	712	1.10	0.92-1.30	719	1.15	0.97-1.37	1,707	1.27	1.14-1.41	1,721	1.21	1.09-1.34
	None (ref.)	5,191	1.00		5,206	1.00		7,045	1.00		7,083	1.00	
	Customer contact												
	Yes, daily	2,611	0.62	0.54-0.72	1,895	0.73	0.63-0.84	6,634	0.60	0.54-0.66	5,281	0.85	0.78-0.93
	Yes, occasionally	1,576	0.69	0.59-0.81	1,285	0.75	0.65-0.88	2,111	0.79	0.70-0.90	2,018	0.89	0.80-1.00
	Yes, rarely	1,267	0.84	0.72-0.99	1,580	0.91	0.79-1.04	1,608	0.84	0.74-0.95	1,932	0.93	0.84-1.04
	No (ref.)	1,327	1.00		2,029	1.00		2,102	1.00		3,276	1.00	

*Controlled direct effects (CDE): Odds ratios (OR) with 95% confidence interval (CI) derived from proportional odds models with adjustment for sex (male; female), age (continuously per 10 years), education (high; medium; low), employment relationship (permanent; fixed-term; temporary; civil servant; self-employed), weekly working hours (full time, ≥35h; 30-35h; 20-<30h; <20h), loneliness at work (none/mild; moderate/severe), chronic work-related stress (no; yes), overcommitment to work, work-privacy conflicts (very high/high; medium; low/very low)

Separate models were calculated for each occupation or occupational risk factor.

Table S5.9 Odds ratios for increased depression and anxiety symptoms by occupational classification and occupational risk factors with **stratification by age**, controlled direct effects

		t1 (November 2023)			t0 (Omicron wave 2022)			t1 (November 2023)			t0 (Omicron wave 2022)		
		N	OR	95% CI	N	OR	95% CI	N	OR	95% CI	N	OR	95% CI
		Young people (≤50 years at t1, n=9,988)						Old people (>50 years at t1, n=10,709)					
**Occupations**													
**S1**	**Production of goods**	1,269	1.09	0.96-1.23	1,272	0.97	0.86-1.10	1,499	1.10	0.97-1.24	1,512	1.00	0.89-1.13
S11	Agriculture, forestry and horticulture	115	0.96	0.66-1.40	116	1.02	0.71-1.45	169	1.26	0.93-1.72	171	1.31	0.98-1.77
S12	Manufacturing	282	1.32	1.04-1.67	283	1.15	0.91-1.46	292	1.09	0.86-1.39	298	1.10	0.86-1.39
S13	Occupations concerned with production technology	464	1.06	0.88-1.29	465	0.87	0.72-1.06	433	1.11	0.90-1.38	437	0.97	0.79-1.20
S14	Building and interior construction	408	0.96	0.78-1.18	408	0.98	0.80-1.19	605	1.00	0.84-1.19	606	0.89	0.74-1.06
**S2**	**Personal services**	3,584	0.69	0.63-0.75	3,577	0.83	0.76-0.91	3,358	0.76	0.70-0.84	3,393	0.90	0.82-0.99
S21	Food industry, gastronomy and tourism	193	0.85	0.64-1.14	192	0.90	0.68-1.19	159	0.99	0.72-1.37	163	0.87	0.63-1.20
S22	Medical and non-medical health care	1,824	0.66	0.59-0.73	1,829	0.84	0.75-0.93	1,606	0.86	0.77-0.97	1,622	1.00	0.89-1.12
S23	Service in social sector and cultural work	1,567	0.89	0.79-1.00	1,556	0.93	0.83-1.03	1,593	0.77	0.69-0.87	1,608	0.87	0.78-0.97
**S3**	**Business administration and related services**	3,077	1.23	1.13-1.35	3,086	1.12	1.03-1.22	3,490	1.13	1.03-1.23	3,518	1.07	0.98-1.17
S31	Commerce and trade	740	1.06	0.91-1.23	746	1.02	0.88-1.18	1,050	0.97	0.85-1.11	1,053	0.98	0.86-1.12
S32	Business management and organisation	428	0.97	0.80-1.18	429	0.90	0.74-1.08	512	1.04	0.86-1.25	516	0.91	0.76-1.10
S33	Business related service occupations	1,909	1.31	1.18-1.45	1,911	1.19	1.08-1.32	1,928	1.20	1.07-1.33	1,949	1.15	1.04-1.28
**S4**	**Service in the IT-sector and the natural sciences**	1,102	1.23	1.08-1.41	1,105	1.18	1.03-1.34	781	1.05	0.90-1.24	786	0.99	0.85-1.16
S41	Service in the IT-sector and the natural sciences	1,102	1.23	1.08-1.41	1,105	1.18	1.03-1.34	781	1.05	0.90-1.24	786	0.99	0.85-1.16
**S5**	**Other commercial services**	504	1.13	0.93-1.36	506	1.02	0.85-1.23	646	1.23	1.04-1.45	650	1.11	0.94-1.31
S51	Safety and security	241	0.98	0.74-1.30	243	0.97	0.74-1.28	209	1.06	0.79-1.43	209	1.01	0.75-1.35
S52	Traffic and logistics	233	1.31	1.00-1.71	234	1.06	0.81-1.37	387	1.28	1.04-1.59	389	1.19	0.96-1.47
S53	Cleaning services	30	0.94	0.47-1.88	<30			50	1.35	0.76-2.41	52	0.94	0.53-1.67
**Other occupational risk factors**													
	Occupational SARS-CoV-2 infection risk												
	Very High	1,709	0.66	0.59-0.74	1,713	0.85	0.76-0.95	1,498	0.85	0.75-0.96	1,512	1.02	0.90-1.15
	High	702	0.92	0.78-1.08	701	0.99	0.85-1.16	746	0.86	0.73-1.00	750	1.04	0.89-1.21
	Probable	1,179	1.20	1.06-1.37	1,183	1.14	1.01-1.30	1,240	1.20	1.05-1.36	1,257	1.18	1.05-1.34
	None (ref.)	5,946	1.00		5,949	1.00		6,290	1.00		6,340	1.00	
	Customer contact												
	Yes, daily	4,366	0.56	0.50-0.63	3,539	0.77	0.69-0.85	4,879	0.66	0.58-0.74	3,637	0.86	0.77-0.96
	Yes, occasionally	1,733	0.72	0.63-0.82	1,429	0.81	0.71-0.91	1,954	0.79	0.69-0.91	1,874	0.87	0.77-0.98
	Yes, rarely	1,491	0.80	0.70-0.92	1,610	0.91	0.81-1.03	1,384	0.90	0.77-1.05	1,902	0.94	0.83-1.06
	No (ref.)	1,913	1.00		2,922	1.00		1,516	1.00		2,383	1.00	

*Controlled direct effects (CDE): Odds ratios (OR) with 95% confidence interval (CI) derived from proportional odds models with adjustment for sex (male; female), age (continuously per 10 years), education (high; medium; low), employment relationship (permanent; fixed-term; temporary; civil servant; self-employed), weekly working hours (full time, ≥35h; 30-35h; 20-<30h; <20h), loneliness at work (none/mild; moderate/severe), chronic work-related stress (no; yes), overcommitment to work, work-privacy conflicts (very high/high; medium; low/very low)

Separate models were calculated for each occupation or occupational risk factor.

Table S5.10. Odds ratios for increased depression and anxiety symptoms by occupational classification and occupational risk factors with **stratification by education**, controlled direct effects

		t1 (November 2023)			t0 (Omicron wave 2022)			t1 (November 2023)			t0 (Omicron wave 2022)		
		N	OR	95% CI	N	OR	95% CI	N	OR	95% CI	N	OR	95% CI
		Low education (no university degree, n=7,625)						High education (university degree, n=13,123)					
**Occupations**													
S1	Production of goods	1,036	1.18	1.02-1.36	1,043	1.04	0.91-1.20	1,732	1.04	0.93-1.16	1,741	0.95	0.85-1.06
S11	Agriculture, forestry and horticulture	112	1.38	0.95-1.99	113	1.36	0.95-1.95	172	0.99	0.73-1.35	174	1.05	0.78-1.41
S12	Manufacturing	299	1.20	0.95-1.52	303	1.11	0.88-1.40	275	1.18	0.92-1.51	278	1.12	0.87-1.43
S13	Occupations concerned with production technology	287	1.18	0.93-1.51	290	0.93	0.72-1.19	610	1.04	0.87-1.24	612	0.90	0.76-1.07
S14	Building and interior construction	338	0.99	0.79-1.24	337	0.95	0.76-1.20	675	0.98	0.83-1.16	677	0.91	0.78-1.08
**S2**	**Personal services**	2,227	0.70	0.63-0.78	2,228	0.80	0.71-0.88	4,715	0.72	0.66-0.78	4,742	0.90	0.83-0.97
S21	Food industry, gastronomy and tourism	210	0.87	0.66-1.16	210	0.82	0.62-1.08	142	0.99	0.71-1.39	145	0.98	0.71-1.37
S22	Medical and non-medical health care	1,493	0.70	0.62-0.79	1,499	0.87	0.77-0.98	1,937	0.77	0.69-0.85	1,952	0.93	0.84-1.03
S23	Service in social sector and cultural work	524	0.85	0.71-1.03	519	0.78	0.65-0.93	2,636	0.81	0.74-0.89	2,645	0.93	0.85-1.02
**S3**	**Business administration and related services**	2,769	1.10	1.00-1.22	2,781	1.10	1.00-1.21	3,798	1.27	1.18-1.38	3,823	1.12	1.04-1.22
S31	Commerce and trade	977	0.90	0.78-1.03	980	0.95	0.83-1.09	813	1.20	1.03-1.39	819	1.08	0.93-1.25
S32	Business management and organisation	315	0.94	0.75-1.18	319	0.91	0.73-1.13	625	1.07	0.91-1.26	626	0.93	0.78-1.09
S33	Business related service occupations	1,477	1.27	1.13-1.43	1,482	1.22	1.08-1.37	2,360	1.27	1.16-1.40	2,378	1.17	1.07-1.29
**S4**	**Service in the IT sector and the natural sciences**	391	1.43	1.16-1.76	392	1.25	1.02-1.54	1,492	1.07	0.95-1.21	1,499	1.04	0.93-1.17
S41	Service in the IT sector and the natural sciences	391	1.43	1.16-1.76	392	1.25	1.02-1.54	1,492	1.07	0.95-1.21	1,499	1.04	0.93-1.17
**S5**	**Other commercial services**	676	1.16	0.98-1.37	677	1.10	0.93-1.29	474	1.16	0.96-1.41	479	0.98	0.81-1.19
S51	Safety and security	203	0.89	0.65-1.23	204	1.03	0.76-1.40	247	1.06	0.81-1.39	248	0.89	0.68-1.17
S52	Traffic and logistics	409	1.27	1.04-1.56	408	1.13	0.92-1.39	211	1.28	0.96-1.70	215	1.09	0.82-1.44
S53	Cleaning services	64	1.17	0.71-1.92	65	1.01	0.62-1.65	16	1.18	0.43-3.19	16	0.93	0.35-2.45
**Other occupational risk factors**													
	Occupational SARS-CoV-2 infection risk												
	Very High	1,403	0.70	0.62-0.80	1,408	0.88	0.78-1.00	1,804	0.75	0.67-0.84	1,817	0.94	0.84-1.05
	High	692	0.84	0.71-1.00	693	0.90	0.77-1.06	756	0.90	0.77-1.04	758	1.09	0.94-1.26
	Probable	986	1.06	0.92-1.23	991	1.14	0.99-1.31	1,433	1.34	1.19-1.51	1,449	1.20	1.07-1.35
	None (ref.)	4,018	1.00	-	4,029	1.00	-	8,218	1.00	-	8,260	1.00	-
	Customer contact												
	Yes, daily	3,592	0.62	0.55-0.71	3,014	0.73	0.65-0.82	5,653	0.59	0.53-0.66	4,162	0.86	0.78-0.95
	Yes, occasionally	1,055	0.80	0.68-0.94	982	0.81	0.69-0.94	2,632	0.73	0.65-0.82	2,321	0.87	0.78-0.97
	Yes, rarely	958	0.89	0.75-1.04	1,107	0.80	0.69-0.93	1,917	0.82	0.72-0.94	2,405	1.01	0.91-1.12
	No (ref.)	1,476	1.00	-	1,978	1.00	-	1,953	1.00	-	3,327	1.00	-

*Controlled direct effects (CDE): Odds ratios (OR) with 95% confidence interval (CI) derived from proportional odds models with adjustment for sex (male; female), age (continuously per 10 years), education (high; medium; low), employment relationship (permanent; fixed-term; temporary; civil servant; self-employed), weekly working hours (full time, ≥35h; 30-35h; 20-<30h; <20h), loneliness at work (none/mild; moderate/severe), chronic work-related stress (no; yes), overcommitment to work, work-privacy conflicts (very high/high; medium; low/very low)

Separate models were calculated for each occupation or occupational risk factor.
